# Evaluation of Plant-Guided Strategies Against Clinical Multidrug-Resistant Pathogens: Preliminary Phytochemical Screening, Antioxidant Capacity, and Antibacterial/Antibiofilm Activity of *Rosa canina* and *Colchicum autumnale* Extracts

**DOI:** 10.3390/antibiotics15050508

**Published:** 2026-05-18

**Authors:** Natalia Vaou, Chrysoula (Chrysa) Voidarou, Evangelia Dimitroulia, Georgios Rozos, Maria Skoufou, Chrysa Saldari, Elisavet Stavropoulou, Georgia Vrioni, Athanasios Tsakris

**Affiliations:** 1 Department of Microbiology, Medical School, National and Kapodistrian University of Athens, 11527 Athens, Greece; nvaou95@gmail.com (N.V.); evidim@med.uoa.gr (E.D.); chrysasaldari@gmail.com (C.S.); elisabeth.stavropoulou@gmail.com (E.S.); gvrioni@med.uoa.gr (G.V.); 2 Department of Agriculture, School of Agriculture, University of Ioannina, 47100 Arta, Greece; xvoidarou@uoi.gr (C.V.); grozos@uoi.gr (G.R.); 3 Laboratory of Hygiene and Environmental Protection, Department of Medicine, Democritus University of Thrace, Dragana, 68100 Alexandroupolis, Greece; mskoufou@yahoo.com

**Keywords:** *Rosa canina*, *Colchicum autumnale*, plant extracts, antimicrobial resistance, MIC/MBC, DPPH/FRAP, antibiofilm activity

## Abstract

**Background/Objectives:** Antimicrobial resistance, an evolutionarily entrenched microbial capacity amplified by extensive antibiotic exposure, has increased the burden of difficult-to-treat infections caused by priority pathogens such as *Klebsiella pneumoniae*, *Pseudomonas aeruginosa* and *Staphylococcus aureus*. In this study, we assessed whether phytochemical-rich extracts from fully ripe *Rosa canina* pseudo-fruits (WF) and fully developed *Colchicum autumnale* flowers (CA) can provide combined antioxidant, antibacterial, and antibiofilm effects against multidrug-resistant clinical isolates. **Methods:** Plant materials were processed using seven extraction systems spanning non-polar to polar conditions (*n*-hexane, ethyl acetate, n-butanol, aqueous, 40% ethanol, 60% ethanol, and enzyme-assisted hydrolysis). Fractions were quantified for total phenolics, flavonoids, and tannins, evaluated for antioxidant capacity (DPPH and FRAP), tested for antibacterial activity (disc diffusion and MIC/MBC), and assessed for inhibition of early biofilm attachment. Differences among extraction methods and fractions were analyzed using standard comparative statistics (group comparisons across solvents/fractions), and relationships between chemical composition and bioactivity were examined using correlation-based analysis. **Results:** Extraction strategy emerged as the main determinant of bioactivity across endpoints. The WFE/ENZ fraction maximized phytochemical recovery (TPC 203.34 ± 11.55 mg GAE/g DW; TFC 35.67 ± 3.06 mg QE/g DW; TTC 53.00 ± 2.65 mg TAE/g DW) and showed strong antioxidant performance (DPPH IC_50_ 33.60 ± 0.02 μg/mL; FRAP A_700_ 1.90 ± 0.010 at 250 μg/mL). Antibacterial effects were strongest in polar fractions, particularly hydroethanolic and enzyme-assisted extracts, while *n*-hexane fractions were consistently weakest. Across eight clinical isolates and three reference strains, MIC values ranged from 0.04875 to 6.25 mg/mL for WF extracts and 0.0975–12.5 mg/mL for CA extracts. In the biofilm model, suppression of early attachment was most consistent for CAE/E60–ENZ and WFE/E40–E60–ENZ fractions. **Conclusions:** Correlation analysis indicated that antibacterial potency aligned primarily with flavonoid levels in *R. canina* pseudo-fruits and with tannin content in *C. autumnale* material. Overall, these results support hydroethanolic and enzyme-assisted extraction as rational strategies to enrich polyphenol-dense fractions with convergent antioxidant, antibacterial, and antibiofilm activity, reinforcing plant-derived matrices as a structured discovery space for developing complementary antimicrobial solutions beyond conventional antibiotics. Notably, this is among the first studies to evaluate the antibacterial potential of *C. autumnale* plant material in this context and to comprehensively assess *R. canina* pseudo-fruit extracts against multidrug-resistant clinical.

## 1. Introduction

Humans exist as holobionts within dense, structured microbial consortia that colonize the gut, respiratory tract, skin, and urogenital mucosa, where community metabolism and host immune calibration maintain a dynamic equilibrium rather than sterility [1,2,3]. Importantly, antimicrobial molecules and the corresponding resistance determinants long predate clinical medicine since resistance genes evolved as ecological survival traits within hostile environments over a long time [4,5]. The scale however and the connectivity of contemporary human-driven selection present an unprecedented historical novelty: antibiotics and other biocides are now prevalent across healthcare, prophylaxis-intensive settings, livestock production, and crop protection, constantly enriching resistant subpopulations within linked human–animal–environment reservoirs and enabling rapid global dissemination via travel and trade [6,7]. Consequently, antimicrobial resistance (AMR) is at the same time an ancient evolutionary biological capacity and an anthropogenically accelerated process, an ecological feedback from microbial networks indicating that current antimicrobial patterns destabilize host–microbe equilibria [8,9].

In this context, the rise in AMR constitutes an internationally recognized “silent” but salient pandemic: it is already narrowing therapeutic options and measurably undermining the clinical reliability of first-line antibacterial therapeutic regimens, thereby threatening the durability of modern treatment protocols. Recent EU surveillance further shows diverging trajectories in invasive disease: methicillin-resistant *Staphylococcus aureus* (MRSA) bloodstream infections declined to 4.48/100.000 in 2024 (−20.4% vs. 2019), while third-generation cephalosporin-resistant *Escherichia coli* increased to 11.03/100.000 (+5.9%) and carbapenem-resistant *Klebsiella pneumoniae* rose to 3.51/100.000 (+61.0%), while carbapenem-resistant *Pseudomonas aeruginosa* remains above 10% of invasive isolates in many European countries, highlighting accelerating pressure from carbapenem resistance [10]. WHO surveillance data indicate that approximately one in six laboratory-confirmed bacterial infections is resistant to standard antibiotics, rising to nearly one in three in some regions [11]. Global evidence signifies that bacterial AMR has moved beyond a theoretical risk into a quantifiable mortality driver: in 2021, an estimated 1.14 million deaths were directly attributable to bacterial AMR, with 4.71 million deaths associated overall, and mortality rising most sharply in older adults [12]. This global signal is mirrored at the regional level; in the EU/EEA alone, more than 35,000 deaths per year are estimated to be directly attributable to infections caused by antibiotic-resistant bacteria, confirming a sustained public-health burden rather than a sporadic treatment failure [10]. In parallel, a synthetic analysis across 187 countries (2000–2024) indicates an approximate 43% global increase in multidrug-resistant infections, with the steepest growth in healthcare-associated infections, underscoring a continuous selection and transmission pressure in both clinical and community settings [13].

Together, these data identify AMR as a major factor of antibacterial treatment failure and point to a deeper ecological shift: the human microbiota increasingly functions as a widespread, gene-rich resistome that has been disseminated across vast host populations offering transferable resistance determinants to opportunistic and invasive pathogens [14,15,16]. The underlying mechanism of this expansion reflects the capacity of microbiota-embedded resistance genes to persist as a stable reservoir and to be rapidly mobilized via horizontal gene transfer under antibiotic exposure, enabling thus the observed accelerated emergence and fixation of resistant phenotypes in clinical pathogens.

Displaying this epidemiological data, the 2024 WHO Bacterial Priority Pathogens List elevates carbapenem-resistant *K. pneumoniae* to the “critical” tier and prioritizes *P. aeruginosa* and *S. aureus*, emphasizing that the principal hazard is the layered resistance architecture these pathogens deploy—β-lactamases/carbapenemases alongside permeability loss, efflux upregulation, and target alteration, and (for MRSA) PBP2a-mediated β-lactam escape—rather than a mere presence [17,18,19,20]. With a constrained antibiotic pipeline from the pharmaceutical industry and the eventually unavoidable resistance to each new agent, the contemporary AMR control strategy increasingly emphasizes mechanism-guided combinations and ecologically informed interventions that reduce selection pressure and expand therapeutic functionality beyond the “more of the same” antibiotic discovery [3,21]. New antibacterial substances with novel chemical structure and modes of action are urgently needed [22,23,24].

Phytochemicals are plant-derived secondary metabolites that encompass diverse structural and functional classes, including alkaloids, phenolics, coumarins, and terpenoids, many of which demonstrate in vitro activity against clinically relevant pathogens [25,26,27,28]. Unlike most conventional antibiotics that act through a dominant single target, phytochemicals often exert antibacterial effects through multi-site interactions, disrupting membranes, attenuating protein synthesis and other intracellular processes, and interfering with biofilm development, efflux-mediated tolerance, and quorum sensing. Their capacity to engage multiple bacterial components, often described as molecular promiscuity, provides a mechanistic rationale for antibacterial activity even when classical resistance determinants compromise target-specific agents and supports their consideration as complementary antimicrobials or adjuvants [29].

Within this conceptual framework, the present study focuses on highly resistant clinical isolates of *K. pneumoniae*, *P. aeruginosa* and *S. aureus* recovered from bloodstream and respiratory infections. The isolates display a broad loss of susceptibility to first-line antibacterial agents and mirror the resistant phenotypes increasingly encountered in intensive-care and high-dependency environments, where therapeutic options are frequently narrowed to last-line regimens. Using these bacteria as clinically relevant targets, we evaluated solvent-partitioned plant extracts as structured phytochemical reservoirs from two species: (i) *Rosa canina* L. (dog rose) pseudo-fruits (rose hips; WF) and (ii) *Colchicum autumnale* L. flowers (CA), collected from mountainous regions of Epirus, Greece. Rather than foregrounding ethnobotanical use, we treated the extracts as chemically defined matrices enriched in phenolics, flavonoids, tannins, and other bioactive compounds and cross-examined how extraction-driven composition correlated with antioxidant performance and antibacterial/antibiofilm outcomes.

Accordingly, the central question was whether plant-derived phytochemicals, can meaningfully inhibit growth and disrupt early biofilm establishment in real-world multidrug-resistant (MDR) isolates belonging to major priority pathogens. By integrating quantitative phytochemical profiling with complementary redox- and membrane-relevant assays, alongside comprehensive antibacterial testing that includes antibiofilm endpoints, this study aimed to define plant-guided intervention signatures with potential to complement conventional antibiotics and contribute to strategies that mitigate the escalating burden of antimicrobial resistance.

## 2. Results

For all subsequent analyses, solvent-derived fractions from *R. canina* pseudo-fruits are referred to as WF extracts and those from *C. autumnale* flowers as CA extracts. To distinguish both plant source and extraction system, each sample is denoted by a two-part code: WFE/ or CAE/ followed by the solvent used. Specifically, *n*-hexane, ethyl acetate, n-butanol, water, 40% (*v*/*v*) ethanol, 60% (*v*/*v*) ethanol and enzymatic extraction are coded as n-H, EtOAc, n-B, A, E40, E60 and ENZ, respectively. Thus, for example, WFE/E60 designates the 60% hydroethanolic extract of *R. canina* pseudo-fruits and CAE/n-B the n-butanol extract of *C. autumnale* flowers. These codes/abbreviations are used consistently throughout the Results and Discussion sections without further elaboration.

### 2.1. Profiling of Phytochemical Patterns and Evaluation of the Antioxidant Capacity

**Figure 1:**
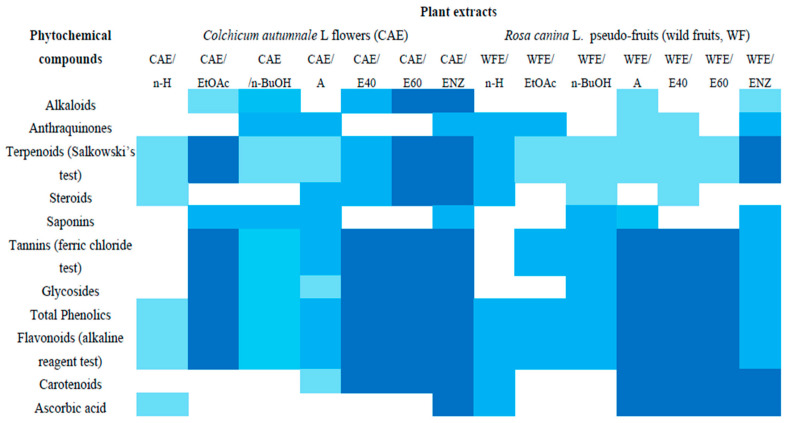
Heatmap summarizing the qualitative screening of major phytochemical classes in the examined extracts. Colour intensity reflects the semi-quantitative detection score for each compound class–extract combination, with higher values indicating stronger phenotypic evidence of the corresponding constituent.

In the present study, all solvent-derived fractions from *C. autumnale* flowers and *R. canina* pseudo-fruits were subjected to a panel of classical qualitative assays to delineate the main classes of secondary metabolites potentially responsible for the observed bioactivities. The semi-quantitative outcomes of these tests are summarized as a heatmap (Figure 1), where gradations from light to dark blue represent increasing detection intensity for each compound class within a given fraction. Figure 1 clearly illustrates the impact of solvent polarity on the extraction profile, revealing both solvent-dependent and plant-dependent patterns. Phenolic compounds and flavonoids were detected in all fractions, indicating broad extractability across the solvent systems employed, whereas tannins were present only at trace or negligible levels in the n-hexane fractions of both *C. autumnale* flowers and *R. canina* pseudo-fruits.

Table 1 presents the total phenolic (TPC), total flavonoid (TFC), and total tannin (TTC) contents of the solvent-partitioned fractions obtained from *C. autumnale* flowers and *R. canina* pseudo-fruits. Across extracts within each plant source, all three phytochemical classes differed significantly as a function of the solvent (Kruskal–Wallis, *p* < 0.05; different superscript letters denote significant pairwise differences). For *C. autumnale*, the n-butanol fraction showed the highest recovery of all three classes (TPC 50.33 ± 2.52 mg GAE/g DW; TFC 24.67 ± 4.04 mg QE/g DW; TTC 20.00 ± 1.00 mg TAE/g DW); whereas, the n-hexane fraction consistently contained the lowest levels (TPC 5.00 ± 1.00; TFC 5.34 ± 0.58; TTC 1.00 ± 0.20). The remaining extracts displayed intermediate values depending on the compound class. For *R. canina*, the enzymatic extract exhibited the greatest enrichment across all three classes (TPC 203.34 ± 11.55 mg GAE/g DW; TFC 35.67 ± 3.06 mg QE/g DW; TTC 53.00 ± 2.65 mg TAE/g DW). In contrast, the n-hexane and ethyl acetate fractions generally showed comparatively low concentrations, particularly for tannins and flavonoids, while hydroethanolic and aqueous fractions yielded intermediate-to-high values depending on the endpoint.

**Figure 2:**
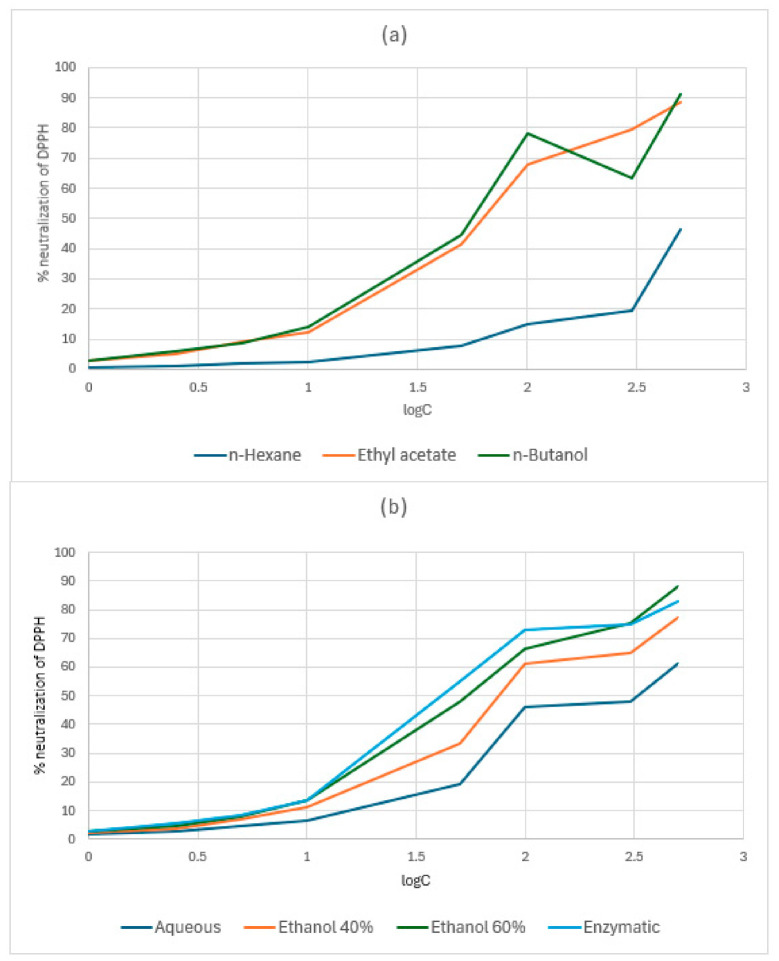
(**a**): Antioxidant (DPPH Radical-Scavenging) Activity of *C. autumnale* extracts obtained with non-polar and medium-polar solvents; (**b**): DPPH Radical-Scavenging Activity of *C. autumale* extracts obtained with polar and enzymatic extraction Systems.

**Figure 3:**
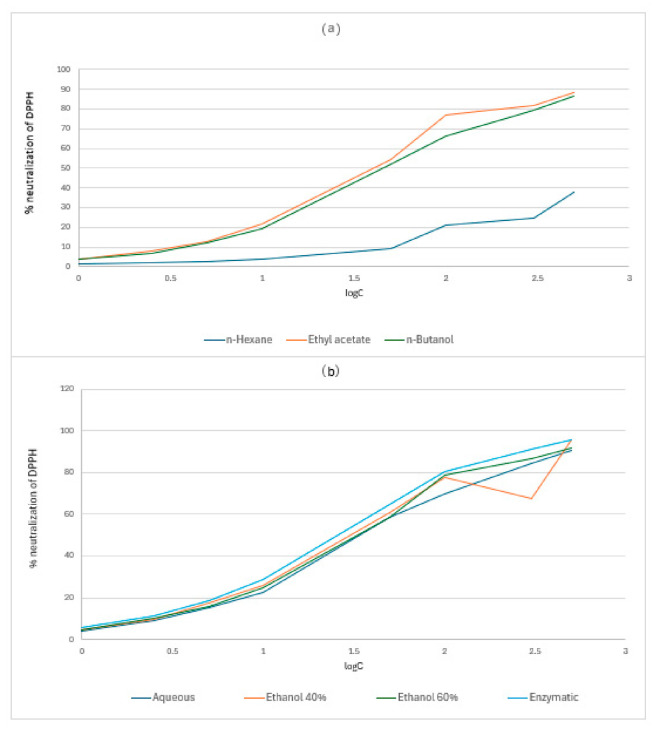
(**a**): Antioxidant (DPPH Radical-Scavenging) activity of *R. canina* extracts obtained with non-polar and medium-polar solvents; (**b**): DPPH Radical-Scavenging activity of *R. canina* extracts obtained with polar and enzymatic extraction systems.

The DPPH assay demonstrated robust, concentration-dependent radical neutralization for all extracts from *C. autumnale* flowers (CA) and *R. canina* pseudo-fruits (WF) (Supplementary Material File S1; Figure 2 and Figure 3). For *C. autumnale* (CA), scavenging capacity was consistently greatest in the medium-to-polar extracts. The n-butanol and ethyl acetate extracts displayed the highest high-dose efficacy, reaching 91.2 ± 1.4% and 88.3 ± 1.4% neutralization at 500 μg/mL, respectively. Hydroethanolic extracts and the enzymatic extract also achieved high inhibition at 500 μg/mL (77.4–87.9% for ethanol 40–60% and 82.9 ± 0.9% for enzymatic), whereas the non-polar n-hexane extract remained comparatively weak across the full dose range, peaking at 46.1 ± 1.1% at 500 μg/mL. Collectively, these patterns indicate that the principal DPPH-quenching constituents in CA are preferentially recovered into medium/high-polarity systems rather than non-polar media. For *R. canina* (WF), the dose–response curves were generally shifted toward higher activity, with multiple extracts approaching or exceeding ~90% neutralization at 500 μg/mL. The enzymatic and ethanol 40% extracts were the most effective at 500 μg/mL (95.6 ± 0.5% and 95.5 ± 0.6%, respectively), while the aqueous extract also showed strong high-dose activity (90.8 ± 0.9% at 500 μg/mL). As in CA, n-hexane showed the lowest scavenging capacity throughout the series (37.9 ± 1.1% at 500 μg/mL), reinforcing the limited contribution of non-polar extracts to DPPH neutralization.

IC_50_ estimates (Table 2) corroborated the dose–response data by quantifying extract potency. In both plant sources, *n*-hexane failed to reach 50% inhibition at the maximum tested concentration (>500 μg/mL), confirming minimal scavenging potency. In CA, the enzymatic extract exhibited the highest potency (IC_50_ 38.47 ± 1.37 μg/mL), while the aqueous extract was substantially less potent (IC_50_ 333.75 ± 6.44 μg/mL). In WF, the enzymatic extract again showed the lowest IC_50_ (33.60 ± 0.45 μg/mL), closely followed by ethanol 40% (37.41 ± 0.33 μg/mL) and ethanol 60% (39.55 ± 0.31 μg/mL).

**Figure 4:**
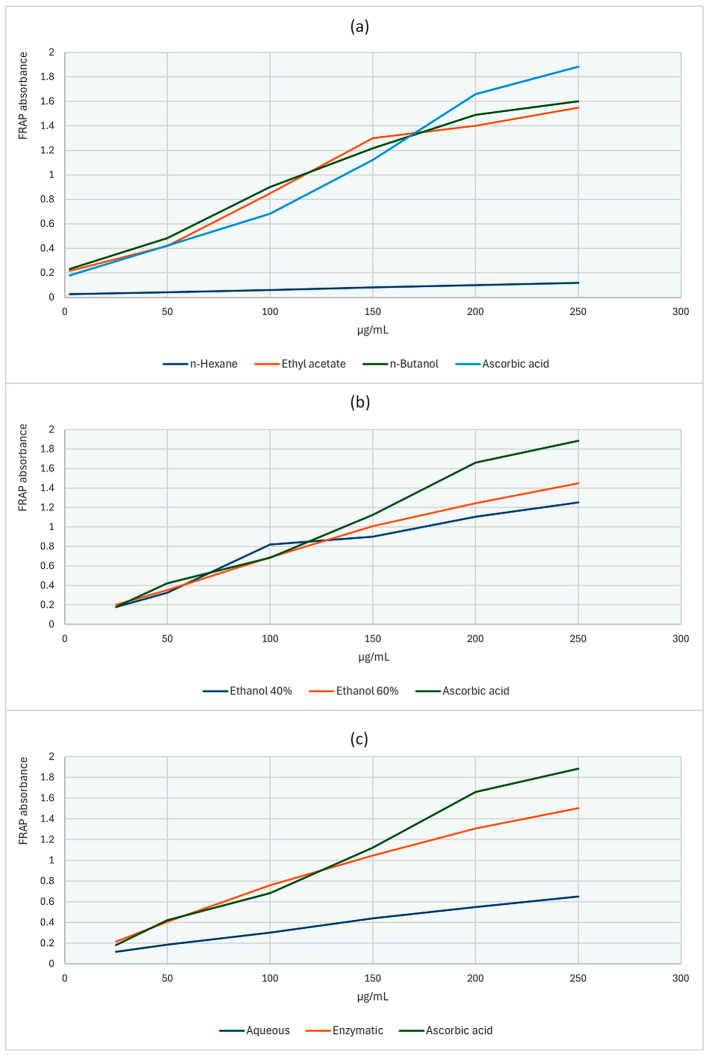
(**a**) Ferric-reducing antioxidant power (FRAP), expressed as absorbance values, of *C. autumnale* extracts obtained with non-polar, medium-polar solvents and reference agent; (**b**) ferric-reducing antioxidant power (FRAP), expressed as absorbance values, of *C. autumnale* extracts obtained with polar solvents and reference agent; (**c**) ferric-reducing antioxidant power (FRAP), expressed as absorbance values, of *C. autumnale* extracts obtained with polar solvent, polar, enzymatic extraction systems and reference agent.

**Figure 5:**
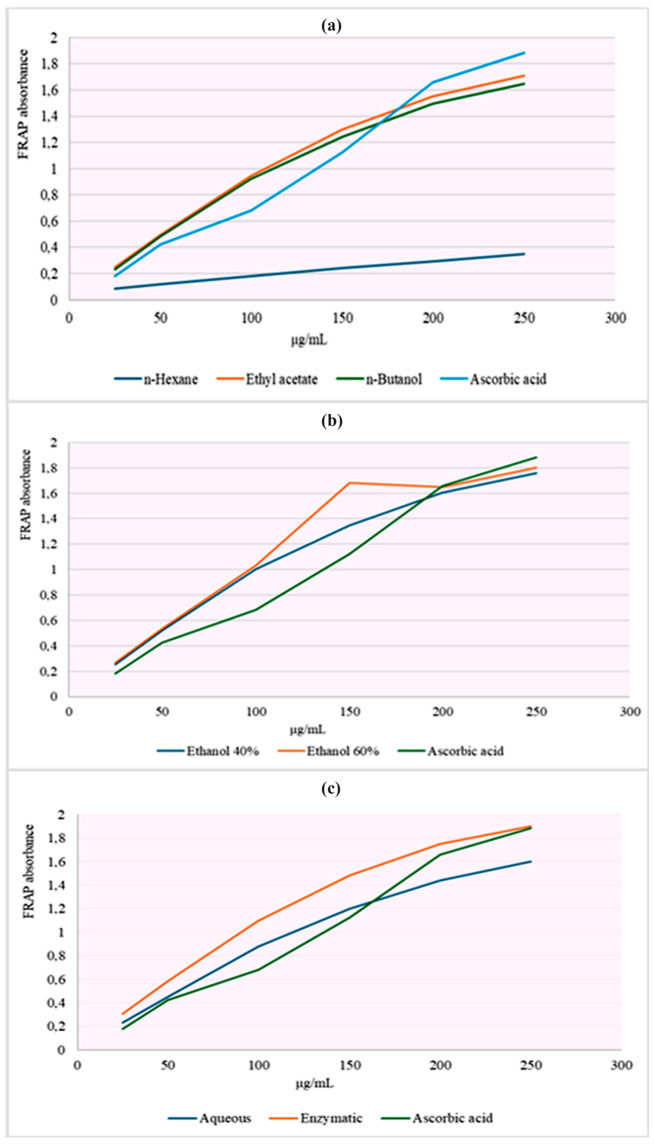
(**a**) Ferric-reducing antioxidant power (FRAP), expressed as absorbance values, of *R. canina* extracts obtained with non-polar, medium-polar solvents and reference agent; (**b**) ferric-reducing antioxidant power (FRAP), expressed as absorbance values, of *R. canina* extracts obtained with polar solvents and reference agent; (**c**) ferric-reducing antioxidant power (FRAP), expressed as absorbance values, of *R. canina* extracts obtained with polar solvent, polar, enzymatic extraction systems and reference agent.

The ferric-reducing antioxidant power (FRAP) assay probes the capacity of an extract to transfer electrons and reduce Fe^3+^ to Fe^2+^ and is therefore widely used as an indirect measure of electron-donating and, by extension, antioxidant/radical-scavenging potential. Consistent with the extract-dependent trends observed in the DPPH assay, the FRAP assay likewise showed pronounced solvent- and concentration-driven differences in antioxidant performance, reflecting variability in electron-donating capacity among the extracts (Supplementary Material File S2; Figure 4 and Figure 5). Across both plant sources, FRAP absorbance increased with concentration, and within each plant source significant differences among extracts were detected at each concentration (Kruskal–Wallis, *p* < 0.05; different superscript letters), confirming that extraction chemistry strongly modulates reducing power.

For *C. autumnale* (CA), the *n*-hexane extract exhibited consistently low reducing power across the entire range and remained minimal at 250 μg/mL (A ≈ 0.12). In contrast, medium-to-polar extracts showed substantially higher FRAP responses at the upper concentrations, with the n-butanol fraction producing the highest terminal absorbance (A ≈ 1.60 at 250 μg/mL) and ethyl acetate also performing strongly (≈1.55). Hydroethanolic and enzymatic extracts displayed intermediate-to-high reducing power (e.g., ethanol 60% ≈ 1.44; enzymatic ≈ 1.50 at 250 μg/mL), whereas the aqueous extract reached a lower plateau (≈0.65), indicating comparatively limited enrichment of reductant-active constituents in water.

For *R. canina* (WF), reducing power was generally higher across extracts at equivalent concentrations and, as in DPPH, the strongest responses were associated with enzymatic and hydroethanolic extraction systems. At 250 μg/mL, the enzymatic extract achieved the highest absorbance (A ≈ 1.90), followed by ethanol 60% (≈1.80) and ethanol 40% (≈1.70–1.75), while ethyl acetate and n-butanol extracts also remained high (≈1.65–1.71). The *n*-hexane extract was again the weakest (≈0.35), underscoring the limited reducing capacity of non-polar extracts. The reference antioxidants (gallic acid and ascorbic acid) reached ~1.88–1.89 at 250 μg/mL, placing the WF enzymatic extract at a comparable—or marginally higher—reducing capacity under these assay conditions.

In Table 3, FRAP results are expressed as EC_50_ (μg/mL), where lower values indicate stronger ferric-reducing capacity. Overall, WF fractions displayed lower and more tightly clustered EC_50_ values (~100–106 μg/mL for E40, E60, ENZ, EtOAc, aqueous, and n-BuOH), whereas CA fractions showed higher EC_50_ values and a broader spread across solvents. For CA, the lowest EC_50_ was observed for EtOAc (106.15 ± 0.93 μg/mL), with hydroethanolic and enzymatic extracts in a similar range, while n-H and n-BuOH presented higher EC_50_ values and greater variability. For WF, the lowest EC_50_ values were recorded for E40/ENZ/E60 (~100–101 μg/mL), and the *n*-hexane fraction showed the highest EC_50_ in both plants, consistent with comparatively weaker reducing power under the assay conditions.

### 2.2. Analysis of Antimicrobial Performance of Plant-Derived Extracts and Suppression of Initial Biofilm Attachment

**Figure 6:**
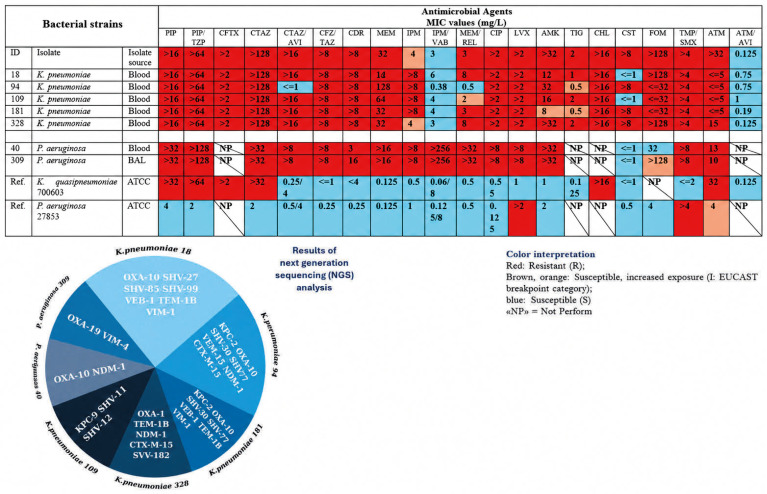
Phenotypically antimicrobial susceptibility profiles of the studied Enterobacterales pathogen isolates and reference strains against common antibiotic agents (Interpretation values according to the EUCAST guidelines). Abbreviations: PIP, Piperacillin; PIP/TZP, Piperacillin/Tazobactam; CFTX, Cefotaxime; CFTAZ, Ceftazidime; CFTAZ/AVI, Ceftazidime/Avibactam; CFZ/TAZ, Ceftolozane/Tazobactam; CFDR, Cefiderocol; MEM, Meropenem; IPM, Imipenem; MEM/VAB, Meropenem/Vaborbactam; IPM/REL, Imipenem/Relebactam; CIP, Ciprofloxacin; LVX, Levofloxacin; AMK, Amikacin; TIG, Tigecycline; CHL, Chloramphenicol; CST, Colistin; FOM, Fosfomycin; TMP-SMX, Trimethoprim/Sulfamethoxazole; ATM, Aztreonam; ATM/AVI, Aztreonam/Avibactam.

To contextualize the antibacterial activity of the investigated plant-derived extracts within a clinically relevant framework, the susceptibility profiles and resistance determinants of the target microorganisms were first characterized. In the present study, the clinical Gram-negative isolates were dominated by multidrug-resistant phenotypes with extensive loss of β-lactam susceptibility. Among *K. pneumoniae* clinical isolates (IDs 18, 94, 109, 181, 328), MIC values (Figure 6) demonstrate extensive compromise of β-lactam activity: piperacillin and piperacillin/tazobactam were uniformly elevated (>16 and >64 mg/L, respectively), third-generation cephalosporins were consistently non-susceptible (cefotaxime > 2 mg/L; ceftazidime > 128 mg/L), and carbapenem activity was markedly reduced (meropenem 32–128 mg/L; imipenem typically >8 mg/L). Cefiderocol MICs were also high (>8 mg/L across isolates), reinforcing the breadth of β-lactam resistance. In contrast, aztreonam/avibactam remained comparatively active across the *K. pneumoniae* set (≤5–15 mg/L for aztreonam with low aztreonam/avibactam MICs of 0.125–1 mg/L), supporting its use as a high-value comparator in this resistant background. “Last-line” susceptibility was isolate-dependent: colistin ranged from ≤1 mg/L (e.g., IDs 18 and 109) to >8 mg/L (e.g., IDs 94, 181, 328), while tigecycline spanned 0.5–2 mg/L. For *P. aeruginosa* (IDs 40 and 309), the MIC profile similarly reflects a severely restricted susceptibility space. Both isolates exhibited very high MICs to multiple antipseudomonal agents (piperacillin > 32 mg/L; piperacillin/tazobactam > 128 mg/L; ceftazidime > 32 mg/L; meropenem > 16 mg/L; imipenem > 8 mg/L), along with poor fluoroquinolone activity (ciprofloxacin and levofloxacin > 8 mg/L). Colistin retained activity in both isolates (≤1 mg/L), highlighting its role as one of the few remaining effective reference drugs in the panel, while cefiderocol activity diverged substantially between isolates (3 vs. 16 mg/L). Crucially, the NGS-derived resistance signatures provide a mechanistic framework that is consistent with these phenotypes, indicating that resistance is not driven by single determinants but by layered β-lactamase repertoires, including carbapenemases (NDM-1, VIM-type enzymes, and KPC-2) alongside ESBL and narrow-spectrum β-lactamases (e.g., CTX-M-15, TEM-1B, SHV variants, and OXA-type enzymes). This gene-level evidence is important for the interpretation of subsequent plant-extract activity, because it implies that any measurable antibacterial or antibiofilm effects are being observed against isolates with multiple, enzymatically mediated routes to β-lactam inactivation (rather than marginal or low-level resistance). For the Gram-positive pathogen, the clinical *S. aureus* isolate (ID 78) exhibited a phenotype consistent with MRSA, as indicated by a positive cefoxitin screen and an oxacillin MIC of 2 mg/L; inducible clindamycin resistance was not detected (D-test negative). In contrast, several agents that remain central to MRSA treatment showed retained in vitro activity, including vancomycin (≤0.5 mg/L), teicoplanin (1 mg/L), linezolid (1 mg/L), daptomycin (1 mg/L), and tigecycline (≤0.12 mg/L). This susceptibility profile provides a clinically meaningful comparator framework for benchmarking the plant-extract activity against a β-lactam–resistant Gram-positive pathogen (Table 4).

Across both plant species, the antibacterial screens converged on a consistent potency landscape when diffusion-derived inhibition zones (Supplementary Material File S3 and S4; Table 5, Table 6, Table 7 and Table 8) were interpreted alongside broth microdilution MICs. In disc diffusion, zone diameters generally expanded as disc content increased (10–100%), yet the magnitude of this dose response varied markedly by extract and bacteria target, indicating that inhibition was determined by extract chemistry and pathogen background, not concentration alone. Importantly, the dataset organized into a reproducible extract hierarchy across the full strain × dose matrix, rather than isolated strain-specific extremes.

For *C. autumnale,* the 60% hydroethanolic extract (E60) was the most consistently active preparation in diffusion assays, dominating the distribution of top-ranked inhibition zones (24/44 largest and 17/44 second-largest outcomes; χ^2^ = 58.8663, *p* < 0.001). This dominance was reflected in the absolute diameters at 100% disc content, where E60 generated pronounced inhibition against both Gram-negative and Gram-positive targets (e.g., *K. pneumoniae* 109: 61.85 ± 0.59 mm; *P. aeruginosa* ATCC 27853: 61.21 ± 0.44 mm; *S. aureus* ATCC 25923: 50.62 ± 0.53 mm). Microdilution confirmed that this diffusion advantage corresponded to intrinsic inhibitory potency: CAE-E60 repeatedly produced the lowest MICs within the set (overall MIC range 0.0975–12.5 mg/L), including 0.0975 mg/mL against *S. aureus* 78, *K. pneumoniae* 109, *K. pneumoniae* 328, and *P. aeruginosa* 40. The enzymatic and n-butanol extracts formed a secondary activity tier—frequently ranking within the top three in diffusion assays—with MICs that were generally low-to-intermediate relative to E60. In contrast, *n*-hexane and (often) aqueous preparations showed limited diffusion-mediated inhibition and correspondingly elevated MICs (*n*-hexane commonly 6.25–12.5 mg/L), consistent with weak growth suppression under the test conditions. A meaningful isolate effect was evident for *K. pneumoniae* 181, which remained comparatively refractory across CAE fractions (best MIC 1.56 mg/L for n-butanol and E60), illustrating that the extract hierarchy is modulated, though not overturned, by strain-specific susceptibility. A parallel alignment between diffusion and MIC endpoints was observed for *R. canina* pseudo-fruits. Diffusion ranking again identified E60 as the most consistently dominant preparation (21/44 largest and 14/44 second-largest zones; χ^2^ = 58.8663, *p* < 0.001), with large inhibition diameters at 100% disc content across multiple organisms (e.g., *S. aureus* 78: 52.01 ± 0.13 mm; *K. pneumoniae* 94: 49.35 ± 0.34 mm). Microdilution corroborated these trends while clarifying the impact of extraction processing: WFE MICs ranged from 0.04875 to 6.25 mg/L, and the lowest values clustered in enzymatic and hydroethanolic preparations. The enzymatic extract achieved MIC 0.04875 mg/L against *S. aureus* 78, while E60 reached MIC 0.04875 mg/L against *S. aureus* ATCC 25923; among Gram-negative organisms, low MICs were frequently observed for E40/E60 (e.g., *K. pneumoniae* 94: 0.0975 mg/L for both E40 and E60; *P. aeruginosa* 40: 0.0975 mg/L for E40). Conversely, n-hexane consistently ranked among the least potent preparations (often ≥1.56–6.25 mg/mL), mirroring its weak diffusion performance. As in the *C. autumnale* dataset, *K. pneumoniae* 181 was the least susceptible isolate under WFE exposure (several extracts at 6.25 mg/L, improving to 3.125 mg/L with E60), underscoring isolate-specific constraints on extract efficacy even under optimized extraction chemistry.

#### Biofilm-Forming Capacity and Inhibition of Initial Biofilm Attachment (Figure 7, Figure 8 and Figure 9)

**Figure 7:**
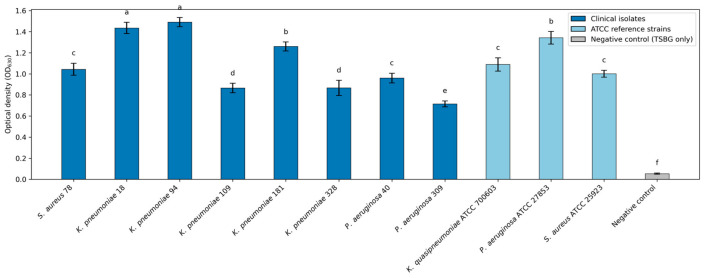
Comparison of biofilm-forming capacity of clinical isolates and ATCC reference strains based on OD_630_ values relative to the negative control (TSBG medium only). Values are means ± SD of triplicate measurements (n = 3). Clinical isolates and ATCC reference strains are displayed in different colours; the negative control (TSBG only) is shown separately. Different letters above bars indicate significant differences among strains (one-way ANOVA with Tukey’s HSD post hoc test, *p* < 0.05); bars sharing at least one letter are not significantly different.

**Figure 8:**
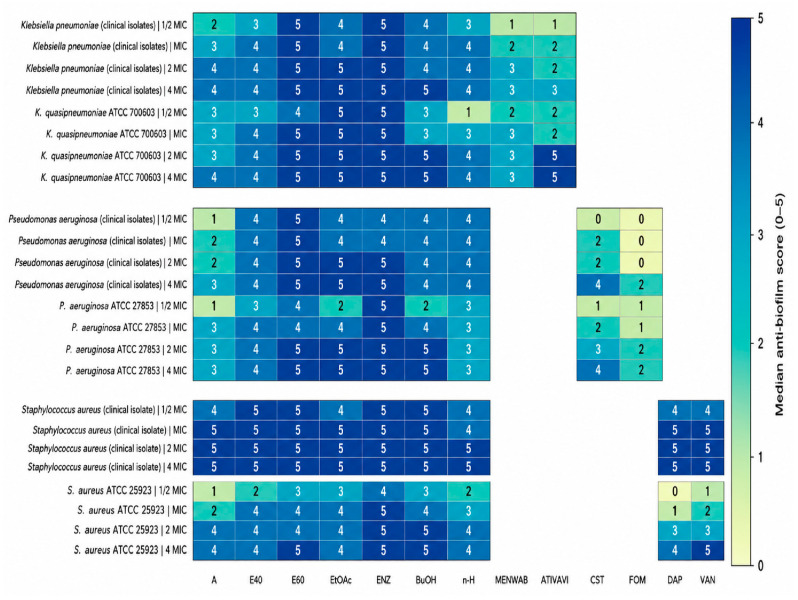
*C. autumnale* flower-derived extracts’ antibiofilm activity (median score, 0–5): comparison of extracts and reference antibiotics across clinical pathogen isolates and ATCC reference strains. Antibiotics were selected based on antimicrobial susceptibility testing, and the MIC values used for 0.5×–4× scaling were set to the relevant EUCAST susceptibility breakpoint for isolates classified as susceptible. Notes: Cell values represent the median antibiofilm score (0–5) derived from three independent replicates (n = 3) for each extract/antibiotic at ½ × MIC, MIC, 2 × MIC, and 4 × MIC. Antibiofilm scores were assigned from the corresponding % biofilm-inhibition values using the predefined categorical scale (0–5). Where applicable, comparisons among treatments within the same bacteria target and concentration were evaluated using a Kruskal–Wallis test (*p* < 0.05) followed by an appropriate rank-based multiple-comparisons post hoc procedure. White cells indicate non-applicable antibiotic–bacteria combinations.

**Figure 9:**
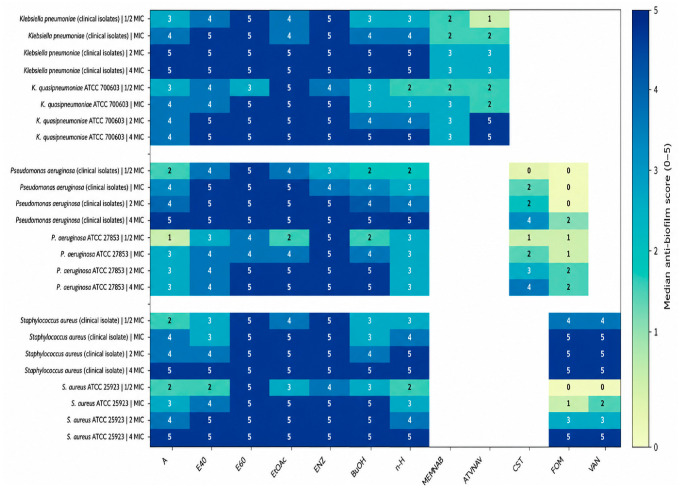
*R. canina* pseudo-fruit-derived extracts antibiofilm activity (median score, 0–5): comparison of extracts and reference antibiotics across clinical pathogen isolates and ATCC reference strains. Antibiotics were selected based on antimicrobial susceptibility testing, and the MIC values used for 0.5×–4× scaling were set to the relevant EUCAST susceptibility breakpoint for isolates classified as susceptible. Notes: Cell values represent the median antibiofilm score (0–5) derived from three independent replicates (n = 3) for each extract/antibiotic at ½ × MIC, MIC, 2 × MIC, and 4 × MIC. Antibiofilm scores were assigned from the corresponding % biofilm-inhibition values using the predefined categorical scale (0–5). Where applicable, comparisons among treatments within the same bacteria target and concentration were evaluated using a Kruskal–Wallis test (*p* < 0.05) followed by an appropriate rank-based multiple-comparisons post hoc procedure. White cells indicate non-applicable antibiotic–bacteria combinations.

Under the biofilm-inducing conditions, all tested bacteria generated quantifiable surface-associated biomass, with OD values consistently above the medium-only control, confirming that both the clinical isolates and ATCC strains expressed a biofilm-forming phenotype in this model (Figure 7). Moreover, baseline biofilm output was not uniform: biomass differed significantly among strains (one-way ANOVA with Tukey’s HSD, *p* < 0.05), indicating a strong isolate-dependent attachment capacity that provides the appropriate context for interpreting antibiofilm performance. Within this set, the most pronounced biofilm formation was recorded for K. pneumoniae isolates, particularly isolates 94 and 18, and for the ATCC dataset, *P. aeruginosa* strain, whereas *P. aeruginosa* 309 consistently exhibited the lowest biomass signal. Building on these baseline phenotypes, both plant sources, *C. autumnale* flower extracts (CAE) and *R. canina* pseudo-fruit extracts (WFE), suppressed initial cell attachment in a concentration-dependent manner across ½ × MIC to 4 × MIC (Figure 8 and Figure 9). The heatmaps condense these outcomes into a 0–5 antibiofilm score derived from percent inhibition (median of triplicate measurements), enabling side-by-side benchmarking of extract performance against reference antibiotics for each organism and exposure level. Across studied bacteria, higher scores became increasingly prevalent at ≥MIC, consistent with progressive impairment of early-stage surface colonization as extract exposure increased. For CAE (Figure 6), antibiofilm activity was structured primarily by extract chemistry: the highest and most consistent scores were concentrated in the more polar preparations, especially the 60% hydroethanolic extract (E60) and the enzymatic extract (ENZ), which repeatedly achieved “very good/excellent” inhibition profiles at ≥MIC against *K. pneumoniae*, *P. aeruginosa*, and *S. aureus*. In contrast, the non-polar *n*-hexane extract showed comparatively weaker and less stable performance, most apparent at ½ × MIC. For WFE (Figure 9), a similar polarity/processing dependence was observed, with robust inhibition concentrated in E40/E60 and ENZ; additionally, the ethyl acetate extract (EtOAc) displayed stronger and more sustained antibiofilm scoring than the corresponding CAE preparation as exposure increased. Within each bacterial target and at each concentration, differences among treatments were assessed using a rank-based Kruskal–Wallis test with post hoc multiple-comparisons procedures (*p* < 0.05).

Table 9 presents a comparative summary of the principal tentatively annotated phytochemicals detected in the four selected extracts (CAE/E60, CAE/ENZ, WFE/E60, and WFE/ENZ). These extracts were prioritized for chromatographic characterization because, across the screening workflow, they provided the most informative combination of phytochemical richness, antioxidant capacity, antibacterial activity, and inhibition of early biofilm attachment, thereby capturing the main chemistry–bioactivity relationships of the study. Compounds are listed according to chemical identity and phytochemical class for comparative purposes and are not arranged by chromatographic elution order. The table is intended to provide a concise overview of cross-extract occurrence patterns among the fractions showing the strongest combined antioxidant, antibacterial, and antibiofilm performance. Detailed UHPLC-DAD-MS/MS annotation data, including retention time, precursor ions, MS/MS fragments, peak-purity values, and annotation criteria, are provided in the Supplementary Material (Supplementary Material Files S5 and S6).

## 3. Discussion

Antimicrobial resistance (AMR) has evolved into a systems-level problem shaped by how antibiotics are prescribed, deployed in food production, and released into the environment—creating persistent selective pressure across connected human, animal, and environmental microbiota and accelerating the emergence and dissemination of MDR and pan-resistant lineages. Under such ecological forcing, “more antibiotics” is not a durable solution: without reducing selection pressure, each new molecule predictably selects for its own obsolescence. A defensible path forward therefore couples stewardship and environmental controls with mechanism-guided combinations and adjuvant strategies that preserve existing agents and expand functional antimicrobial capacity.

Within this framework, plant-derived bioactives represent chemically diverse, evolutionarily refined defence repertoires that may act as direct antibacterials and/or as modulators of virulence and biofilm development, thereby complementing conventional therapy. In the present study, we deliberately juxtaposed two contrasting phytochemical reservoirs—*R. canina* pseudo-fruits and *C. autumnale* flowers—collected in parallel from the same mountainous region (Epirus, Greece) to minimize geographic and phenological confounding. Their extracts were challenged against a stringent set of high-consequence clinical isolates (*K. pneumoniae*, *P. aeruginosa*, *S. aureus*) from bloodstream and respiratory infections, representing CRE-like, MDR/XDR and MRSA phenotypes that compress treatment toward last-line options. This design positions the extracts as mechanistically informative test matrices for evaluating whether complex phytochemical mixtures can inhibit growth and attenuate early biofilm establishment in clinically realistic resistant backgrounds.

### 3.1. Phenotypic Screening of Bioactive Molecules

Qualitative (phenotypic) screening highlights both plant matrices as chemically complex but clearly polyphenol-forward, with flavonoids and total phenolics detected across most extraction systems (Figure 1). In *C. autumnale* flowers, this aligns with plant part (organ)-level evidence that flowers are relatively enriched in phenolic-type metabolites, while alkaloids are more prominent in other organs [30]. In both species, the heatmap (Figure 1) resolves a strong solvent-polarity effect: n-hexane shows the weakest tannin-like signatures, whereas medium-to-polar extracts (e.g., ethyl acetate/alcoholic systems) preferentially recover phenolics/flavonoids/tannins, consistent with prior solvent-comparison work on *C. autumnale* and *R. canina* pseudo-fruit [31,32]. This polarity-driven enrichment provides the key chemical context for downstream antioxidant and antimicrobial/antibiofilm testing [33].

### 3.2. Pattern of Total Phenolics, Flavonoids and Tannins Content

The solvent-partitioning profile of *C. autumnale* flowers in the present study follows the well-established polarity dependence of polyphenol recovery: n-hexane was consistently depleted, whereas medium-to-polar extracts—most notably n-butanol, followed by ethyl acetate and hydroethanolic systems—were enriched in total phenolics, flavonoids and tannins, indicating preferential recovery of flower polyphenols under these conditions. Intermediate extracts also showed selective enrichment among phenolic subclasses, consistent with differential partitioning of non-flavonoid phenolics versus flavonoid/tannin-like constituents. This qualitative ranking matches published solvent comparisons in *C. autumnale*; for example, total phenolics increased from 6.448 ± 0.008 (n-hexane) to 17.3 ± 0.003 (methanol) and peaked at 26.6 ± 0.003 µg GAE/mg dry extract (dichloromethane), with total flavonoids showing the same trend (25.5 ± 0.007 > 6.094 ± 0.003 > 4.773 ± 0.005 µg QE/mg for dichloromethane, methanol and *n*-hexane, respectively) [31]. Alcoholic-versus-aqueous comparisons in flowers likewise support higher phenolic-class recovery in alcoholic preparations [34], while metabolite profiling confirms a phenolic-acid-dominated flower matrix (phenolic acids 2.78 mg/g DW; alkaloids 1.39 mg/g DW) [30]. Although absolute magnitudes differ across protocols and reporting bases, the convergence is robust: non-polar extracts are polyphenol-poor, whereas intermediate-to-polar systems yield higher phenolic and flavonoid recovery in *C. autumnale*.

In the present work, *R. canina* pseudo-fruit extracts displayed strong extraction-chemistry dependence: the enzymatic extract maximized all phytochemical classes (TPC 203.34 ± 11.55 mg GAE/g DW; TFC 35.67 ± 3.06 mg QE/g DW; TTC 53.00 ± 2.65 mg TAE/g DW), whereas *n*-hexane yielded the lowest recoveries (TPC 33.67 ± 3.21; TFC 2.50 ± 0.50; TTC 2.00 ± 0.20 mg eq/g DW). This pattern agrees with enzyme-assisted extraction evidence that cellulolytic pretreatment enhances phenolic release (e.g., Lemoni et al., 2025 [33]: TPC 168.3 ± 1.1 mg GAE/g DM) and with the expected poor partitioning of polar phenolics into non-polar media. Reported rosehip totals vary widely with tissue, genotype, ripening, solvent, and reporting basis; Taneva et al., 2016, [35] reported TPC 55.4–69.4 mg GAE/g DW and high aqueous tannins (38.6 mg/g DW), consistent with our strong aqueous TTC (46.34 mg/g DW), while tissue-level fresh-weight reporting yields lower values (e.g., Grigoriadou et al., 2024 [36] up to 31.20 mg GAE/g FW; Miljković et al., 2024 [37] 2.98 mg GAE/g FW and 1.45 mg CE/g FW). Higher extract-normalized values are also documented (e.g., Ergün et al., 2025 [38]: TPC 141.17 ± 1.12 mg GAE/g; TFC 6.04 ± 0.23 mg QE/g), and interspecific surveys indicate inherent 2–3-fold genotype/species effects (≈10.89–26.49 mg GAE/g across *R. canina* pseudo-fruits), underscoring that absolute magnitudes should be interpreted in light of extraction intensity and normalization. The polarity-driven distribution of phytochemicals provides a coherent framework for interpreting extract composition and bioactivity. In this context, Ivanov et al., 2026, reported that *R. canina* fruit extracts are dominated by flavonols, flavan-3-ols, and procyanidins—including quercetin derivatives and procyanidin dimers—with flavan-3-ols/procyanidins preferentially recovered in aqueous-type preparations, consistent with enrichment of tannin-related pools in polar fractions [39]. Complementary LC-based profiling of dog rose infusions and hydromethanolic extracts repeatedly detects quercetin and rutin together with phenolic acids, while highlighting that absolute totals vary substantially with plant matrix and extraction conditions; therefore, bioactivity should be interpreted primarily in relation to extraction architecture and solvent polarity, rather than by direct comparison of single absolute values across studies [40].

Overall, across both matrices, the results are mechanistically consistent with the literature: solvent polarity and extraction architecture (fractionation and enzymatic pretreatment) largely govern the observed TPC/TFC/TTC pattern by controlling phenolic partitioning and matrix-limited release. The reproducible weakness of *n*-hexane in both *C. autumnale* and *R. canina* versus the superiority of medium-polar systems (n-BuOH for *C. autumnale*; enzymatic/hydroethanolic extracts for *R. canina*) indicates that the dominant phenolic/tannin pools are predominantly polar-to-amphiphilic, and that enzymes can further increase yields by cell-wall depolymerization and improved mass transfer.

- Compound-level profiling of the selected active extracts

Compound-level phytochemical profiling of the selected active extracts further supports the primary quantitative findings for total phenolics, flavonoids, and tannins. As shown in Table 9 and in the supplementary UHPLC-DAD-MS/MS data, WFE/E60, WFE/ENZ, CAE/E60, and CAE/ENZ contained tentatively annotated compounds corresponding to the broad chemical groups measured in the earlier colorimetric assays. The flavonoid fraction was represented by flavonols and flavones such as quercetin, kaempferol, luteolin, rutin, apigenin, and kaempferol/quercetin glycoside-type compounds, as well as flavanone-related compounds such as naringenin and naringenin-7-O-glucoside in *R. canina* extracts. Flavan-3-ol-type compounds, specifically catechin/epicatechin-type flavan-3-ols, were detected in both plant matrices. The phenolic-acid fraction included caffeic, ferulic, coumaric, vanillic, syringic, salicylic, rosmarinic, protocatechuic, and ellagic-acid-related compounds, depending on the plant source and extraction system. Tannin-related constituents were supported mainly by catechin/epicatechin-type flavan-3-ols, procyanidin B-type dimer/procyanidin-type oligomer, and ellagic-acid derivatives detected in the *R. canina* extracts. In *C. autumnale*, the selected extracts also contained colchicine-related tropolone alkaloids, including colchicine, demecolcine, colchicoside, colchiciline, colchiceine, deacetamido-5,6-dihydrocolchicine, and demethylcolchicine/colchiceine-type isomers, indicating an additional non-phenolic phytochemical component characteristic of this plant species. Therefore, Table 9 provides compound-level support that the extraction systems associated with higher total phenolic, flavonoid, and tannin values also recovered chemically diverse representatives of these groups. These findings indicate that the quantitative phytochemical results reflect real enrichment of tentatively annotated bioactive constituents rather than only non-specific colorimetric responses.

### 3.3. The Evaluation of Antioxidant Capacity: DPPH Assay and Ferric-Reducing Assay Power (FRAP)

Antioxidant capacity cannot be assessed by a single assay, as different methods probe distinct reaction pathways. The FRAP assay primarily measures electron-donating (reducing) capacity, while the DPPH assay captures radical-quenching behaviour via electron transfer and/or hydrogen-atom donation. In many plant systems, elevated FRAP and DPPH responses correlate with higher phenolic content, reflecting the diverse redox mechanisms of phenolics—including hydrogen-atom transfer, single-electron transfer, sequential proton loss–electron transfer, and metal chelation. Accordingly, this study integrated antioxidant assays with qualitative phytochemical screening and colorimetric quantification of total phenolics, flavonoids, and tannins. For both plant sources, DPPH results exhibited a clear dose–response relationship and strong dependence on extraction solvent. Non-polar n-hexane extracts showed consistently weaker activity, whereas medium-to-polar extracts displayed higher radical neutralization (Supplementary Material File S1; Table 2; Figure 2 and Figure 3). This solvent-dependent trend aligns with established polyphenol extraction behaviour, wherein hydroxyl-rich compounds preferentially partition into mid-polar and polar solvents.

For *C. autumnale* flower extracts, both assays indicated a robust polarity-driven antioxidant gradient, with mid-polar/polar systems consistently outperforming non-polar extraction. In DPPH, the highest inhibition at 500 μg/mL occurred in n-BuOH (91.17 ± 0.95%), EtOAc (88.26 ± 1.27%), and E60 (87.88 ± 0.37%), followed by ENZ (82.94 ± 0.37%); whereas, n-hexane was lowest (46.08 ± 1.80%). Potency ranking was concordant, with the smallest IC_50_ for ENZ (38.47 μg/mL) and E60/n-BuOH (~43.85–44.65 μg/mL) but much higher values for *n*-hexane (275.85 μg/mL) and aqueous (333.75 μg/mL), indicating poor scavenging efficiency in non-polar and strictly aqueous systems. The FRAP assay mirrored this enrichment logic: at 250 μg/mL, reducing power peaked in n-BuOH (1.603 ± 0.012 A700) and remained high in ENZ (1.503 ± 0.006) and E60 (1.447 ± 0.012), while n-hexane was minimal (0.117 ± 0.012) (Supplementary Material File S2; Table 3; Figure 4). This pattern aligns with the solvent-comparison literature; Hailu et al., 2021, likewise report weak antioxidant recovery in *n*-hexane but stronger DPPH/ABTS responses and higher phenolic/flavonoid levels in more polar extracts, including superior activity in dichloromethane/methanol versus hexane, consistent with antioxidant capacity tracking phenolic-class redox donors under medium-polarity conditions [31]. Flower-targeted studies further support this organ-and-solvent dependence; Suica-Bunghez et al., 2017, reported higher DPPH scavenging in flowers (52.81%) than roots (34.60%) alongside substantial polyphenol/tannin loads in hydroalcoholic preparations [41], while Moroșan et al., 2025, show markedly higher antioxidant signal in alcoholic versus aqueous flower extracts, consistent with the present observation that water alone underperforms relative to alcohol-containing and partition-enriched fractions [34]. Finally, plant part (organ)-resolved metabolite profiling by Dincheva et al., 2025, reports measurable flower phenolic acids (2.78 mg/g DW), providing compositional support that flower tissues contain phenolic-class substrates capable of driving redox assays once efficiently extracted/enriched [30].

For *R. canina* pseudo-fruit (WF), the present study reveals a pronounced extraction-driven antioxidant gradient in both assays, with enzyme-assisted and hydroethanolic systems enriching the dominant redox-active pool. In the DPPH assay, several WF extracts approached near-complete scavenging at 500 μg/mL—ENZ (95.6 ± 0.5%) and E40 (95.5 ± 0.6%), with the aqueous extract also high (90.8 ± 0.9%)—whereas n-hexane remained weak (37.9 ± 1.1%). Potency followed the same hierarchy, with the lowest IC_50_ for ENZ (33.60 ± 0.45 μg/mL) and similarly low values for E40 (37.41 ± 0.33 μg/mL) and E60 (39.55 ± 0.31 μg/mL); notably, n-hexane did not achieve 50% inhibition at the highest tested concentration (>500 μg/mL). The FRAP results were concordant: at 250 μg/mL the greatest reducing power was observed for ENZ (A ≈ 1.90), followed by E60 (≈1.80) and E40 (≈1.70–1.75), while n-hexane remained minimal (≈0.35), indicating limited transfer of electron-donating antioxidants into non-polar media (Supplementary Material File S2; Table 3; Figure 5).

This pattern is supported by multiple studies regarding *R. canina* pseudo-fruits showing that antioxidant capacity tracks extraction architecture and phenolic enrichment. In a phenolic-enrichment workflow, Sabahi et al., 2022, reported a marked potency increase from crude extract to a phenolic-rich fraction (DPPH IC_50_: 476 ± 0.50 → 57 ± 0.57 μg/mL; FRAP IC_50_: 39 ± 0.03 → 7 ± 0.01 μg/mL), supporting the interpretation that lower IC_50_ values in our ENZ/E40/E60 extracts reflect enrichment in redox-active phenolics [42]. Classic solvent comparisons similarly show systematic superiority of polar solvents: Montazeri et al., 2011, reported strong activity for a methanolic fraction (DPPH IC_50_ = 11.58 μg/mL) and described the hexane fraction as least active, matching the reproducible polar ≫ non-polar pattern observed in the present work [32]. Likewise, Taneva et al., 2016, found higher antioxidant equivalents in ethanol-containing extracts (DPPH: 211.5–295.0 mM TE/g DW; FRAP: 309.5–390.1 mM TE/g DW across water/50%/70% ethanol), consistent with the strong performance of our hydroethanolic extracts [35]. At the same time, absolute magnitudes differ across reports because of variation in fruit material (e.g., pulp/seed inclusion), extraction conditions, and assay expression. For example, an ethanolic rosehip extract with DPPH IC_50_ = 156.74 ± 0.56 μg/mL illustrates how protocol differences can yield higher IC_50_ values than those obtained in the measurements of the present study [43]. Tissue/protocol comparisons also indicate moderate potencies for fruit extracts (e.g., DPPH IC_50_ = 56.32 μg/mL) and FRAP expressed as TEAC (0.41 for fruit; higher for galls), underscoring the influence of plant tissue and reporting basis on apparent magnitude [38]. Commodity-style reporting on a fresh/dry fruit basis can further compress antioxidant indices (e.g., DPPH 6.84; FRAP 52.04), emphasizing that cross-study numeric comparisons are most reliable when units and normalization are aligned [37]. Complementary lipid-oriented studies also reinforce the mechanistic interpretation: supercritical CO_2_ rosehip oil extracts can show ABTS activity, whereas Soxhlet n-hexane oil may be inactive, consistent with the weak n-hexane performance reported in the present study dataset [44]. Another study supports external compositional/antioxidant datasets: LC-based profiling work comparing infusions vs. hydromethanolic extracts reported higher DPPH and FRAP for infusions and documented positive correlations between phenolic pools and antioxidant indices (e.g., TPC–FRAP and TFC–DPPH), reinforcing that antioxidant output tracks phenolic/flavonoid enrichment and extraction mode [40]. Finally, Grigoriadou et al., 2024, provide a useful tissue-level benchmark for contextualizing our extract-based results: across Greek *R. canina* germplasm, total phenolics reached 31.20 mg GAE g^−1^ FW at late ripening and DPPH-based antioxidant capacity (Trolox equivalents) reached 23.18 mg Trolox g^−1^ fresh tissue, highlighting the strong effects of genotype, developmental stage, and management regime. Although these outcomes are expressed on a fresh-tissue basis and thus are not numerically comparable to extract-normalized DPPH/FRAP measures, they support the same conclusion that rosehip antioxidant performance reflects both intrinsic fruit biochemistry and the efficiency of the applied extraction/enrichment strategy [36].

The compound-level profile presented in Table 9 provides a stronger chemical basis for interpreting the DPPH and FRAP results. The selected active extracts contained multiple redox-relevant constituents, including flavonoids such as quercetin, kaempferol, luteolin, rutin, catechin, and epicatechin, phenolic and hydroxycinnamic acids such as caffeic, ferulic, coumaric, vanillic, syringic, salicylic, and rosmarinic acids, and tannin-related structures such as procyanidin-type and ellagic-acid-related compounds. These compounds are mechanistically relevant to both assays because phenolic hydroxyl groups can donate hydrogen atoms or electrons and stabilize radical intermediates through aromatic resonance, thereby contributing directly to radical-scavenging activity and ferric-reducing power. This interpretation is consistent with the observed extraction-dependent antioxidant pattern, where medium-polar, hydroethanolic, and enzymatic fractions showed stronger DPPH and FRAP responses than non-polar extracts.

In the *C. autumnale* fractions (CAE/E60, and CAE/ENZ), the detection of colchicine-related tropolone alkaloids provides an additional, but more cautious, layer of interpretation. Previous studies confirm that *Colchicum* species contain mixed phytochemical pools composed of tropolone alkaloids, phenolics, tannins, and flavonoids, with colchicine, demecolcine, colchicoside, and demethyl-colchicine derivatives reported as characteristic alkaloid constituents of *C. autumnale* and other members of the genus *Colchicum* [45]. However, colchicine should not be presented as a major direct antioxidant in the same way as phenolic compounds. Kinetic evidence shows that colchicine and tropolone display only weak radical-scavenging properties at high concentrations, although the tropolone ring can participate in radical-related reactions and may therefore provide limited redox contribution depending on the radical system [46]. Therefore, the antioxidant capacity of CAE/E60 and CAE/ENZ is best explained by the combined enrichment of phenolics, flavonoids, hydroxycinnamic acids, catechin/tannin-related compounds, and colchicine-type alkaloids, with the alkaloid fraction acting mainly as a complementary redox-active component rather than the dominant driver of DPPH and FRAP activity.

### 3.4. The Antimicrobial and Antibiofilm Capacity of the Studied Extracts

The agar disc-diffusion screening of the solvent-partitioned extracts against eight MDR clinical isolates and three ATCC reference strains at disc loadings of 10–100% *v*/*v* is presented in the Supplementary Material section (S3 and S4). This assay was used as an initial comparative screen, since inhibition-zone diameters integrate extract potency with disc loading, strain susceptibility, agar diffusion, solubility, polarity, molecular-size distribution, and crude-extract mixture effects; therefore, the non-linear zone responses were interpreted as screening patterns, whereas MIC/MBC values were used as the main quantitative indicators of antibacterial potency. Across *C. autumnale* fractions, antibacterial activity was clearly extraction-dependent and enriched in medium-polar preparations, with E60 showing the strongest and most reproducible inhibition, followed by n-BuOH and ENZ; the overall diffusion ranking was E60 > n-BuOH > ENZ > EtOAc > E40 > A > n-H (χ^2^ = 58.8663, *p* < 0.001). This hierarchy was supported by positive associations between antibacterial rank and total phenolics, tannins, and flavonoids, especially tannins, whose protein-binding, membrane-interactive, nutrient-sequestering, and enzyme-disrupting properties provide a plausible basis for broad antibacterial effects [47,48]. Finally, our results highlight an important methodological point regarding tannin recovery: although tannins are often reported to be efficiently extracted with hot water or aqueous methanol [49,50,51], our fractionation data indicate preferential enrichment in n-butanol, followed by enzyme-assisted extraction and 60% ethanol, whereas 40% ethanol yielded comparatively lower tannin recovery than expected. This emphasizes that tannin partitioning is not universal but depends on matrix composition, solvent polarity, and extraction design, reinforcing the need to interpret antibacterial activity in the context of extraction architecture rather than assuming class-specific “default” solvents. Inhibition-zone diameters in agar disc-diffusion assays represent a composite screening phenotype, reflecting not only intrinsic antibacterial potency but also agar diffusion, effective availability, extract polarity, solubility, molecular-size distribution, and crude-fraction mixture effects. These factors explain the non-linear dose–response patterns and occasional large inhibition zones observed across disc loadings. Therefore, disc diffusion was interpreted as a comparative readout of extract- and strain-dependent activity, while MIC values were used as the primary concentration-based measure of antibacterial potency.

The MIC profile of *C. autumnale* flower extracts demonstrates a clear extraction-dependent antibacterial effect across all tested microorganisms. MIC values ranged from 0.0975 to 12.5 mg/L, with the strongest and most reproducible inhibition concentrated in the medium-polar fractions, particularly E60, and, depending on the strain, n-BuOH and ENZ. By contrast, n-hexane and aqueous extracts more frequently required higher concentrations, indicating that non-polar and water-only extraction recovered the antibacterial phytochemical pool less efficiently. This pattern was especially evident among the *K. pneumoniae* bloodstream isolates, where most *C. autumnale* fractions inhibited growth at ≤3.125 mg/L, despite the high MICs recorded for several conventional antibiotics. In selected cases, n-BuOH and E60 produced very low MICs of 0.0975 and 0.195 mg/L, respectively, even when considered alongside meropenem/vaborbactam as a last-line comparator. A similar trend was observed for *S. aureus*, where E60 and ENZ reached MICs of 0.0975 mg/mL, and for *P. aeruginosa*, where EtOAc, n-BuOH, and E60 showed MICs below colistin, the closest antibiotic comparator in this assay framework. Collectively, these findings indicate that the antibacterial activity of *C. autumnale* is not uniformly distributed across fractions, but is preferentially recovered by extraction systems capable of concentrating medium-polar bioactive constituents.

Although *C. autumnale* has been recognized as a medicinal plant since classical antiquity and is extensively documented in ethnopharmacological and pharmacological contexts, we were unable to identify prior studies that systematically evaluate its antibacterial activity, meaning that the current study is the first to address the subject. The seeds and the corm of the plant after different preparations have been used for indications such as gout, prostatic hyperplasia, musculoskeletal pain, sleep disturbances, familial Mediterranean fever, myeloid leukemia, and other malignancies [52,53]. The strong historical emphasis on these therapeutic applications, together with the prominence of colchicine-related bioactivity, may have contributed to the relative neglect of *C. autumnale* as a source of antibacterial phytochemicals. Most relevant published studies focus on (i) tropolonic alkaloids (colchicine and congeners) and their pharmacology/toxicity rather than antibacterial endpoints [53], and/or (ii) general phytochemical/antioxidant profiling of hydroalcoholic extracts from *C. autumnale* flowers/roots (polyphenols, tannins, flavonoids, terpenoids) without systematic MIC-based antibacterial evaluation [41].

Within this framework, the MIC/MBC data provide a structured antibacterial readout for solvent-partitioned *C. autumnale* flower extracts and reveal a clear extraction-dependent activity pattern. The strongest inhibition was concentrated in medium-polar fractions, particularly CAE/E60, and, in several strain–extract combinations, n-BuOH and CAE/ENZ, whereas non-polar and purely aqueous preparations were less consistently active. This polarity-dependent behaviour agrees with evidence from related *Colchicum* species, where ethanolic extracts of *C. luteum* showed stronger activity than aqueous preparations against *S. aureus* and *P. aeruginosa* [54], and where *C. speciosum* studies support the antimicrobial contribution of volatile/terpenoid-rich oils and phenolic co-constituents [55]. Importantly, Table 9 shows that CAE/E60 and CAE/ENZ are not single-marker fractions, but chemically plural matrices containing colchicine-related tropolone alkaloids together with phenolic acids, hydroxycinnamic acids, flavonoids, and catechin/tannin-related constituents. This composition provides a mechanistic basis for interpreting the antibacterial and antibiofilm effects as the result of overlapping phytochemical actions rather than as an alkaloid-specific response. Alkaloids are increasingly recognized as antimicrobial natural products capable of affecting microbial viability through cell-wall and membrane disruption, interference with protein synthesis, inhibition of DNA replication, enzyme/metabolic disruption, efflux-pump modulation, antibiofilm activity, and synergy with conventional antimicrobials [56]; this is further supported by Huang et al. 2022, who describe terpenoid, alkaloid, and flavonoid antimicrobial agents and their mechanisms, including membrane damage, quorum-sensing interference, protein/nucleic-acid synthesis inhibition, and synergistic effects with antibiotics [57]. *Colchicine* itself, identified as a *C. autumnale*-derived alkaloid, has been listed among natural alkaloids with antimicrobial relevance, including activity against *Aureobasidium pullulans* with an MFC of 1 µg/mL [56], while colchicine C-ring amine derivatives have shown intrinsic antibacterial activity, with MICs of 16–32 µg/mL, together with antibiofilm effects in MRSA models [58]. However, because colchicine-dominant preparations have well-established toxicity concerns [59], the crude-extract activity should be interpreted cautiously as the outcome of extraction-driven enrichment of complementary phytochemicals, not as the isolated effect of colchicine-type alkaloids alone. In parallel, flavonoids detected in these extracts, including quercetin, kaempferol, luteolin, rutin, catechin, and epicatechin, may contribute through membrane and cell-wall disruption, inhibition of protein and nucleic-acid synthesis, signal-transduction interference, efflux-pump suppression, and reduction in biofilm formation and virulence-factor production [60]. Phenolic and hydroxycinnamic acids, including caffeic, ferulic, coumaric, vanillic, syringic, salicylic, and rosmarinic acids, may further support antibacterial activity by interacting with the microbial envelope, penetrating or accumulating at bacterial membranes, disturbing intracellular pH, inhibiting enzymes, and interfering with intracellular targets [61]. Tannin-related compounds, including catechin/epicatechin derivatives, procyanidin-type oligomers, and ellagic-acid-related structures, may strengthen this activity through protein binding, metal chelation, membrane interaction, nutrient limitation, and disruption of extracellular polymeric matrix organization [47,48,62]. Recent antibiofilm literature further supports this interpretation, showing that natural phytochemicals can inhibit quorum sensing, reduce virulence-factor production, prevent initial adhesion, and weaken extracellular polymeric substances, mechanisms that are directly consistent with the early attachment inhibition observed here [63]. Thus, the activity of CAE/E60 and CAE/ENZ is best explained by multi-component phytochemical assemblies generated by specific extraction systems, with possible additive or synergistic interactions among phenolics, flavonoids, tannin-related structures, and colchicine-related alkaloids acting on both planktonic growth and surface-colonization processes.

Unlike *C. autumnale*, *R. canina* has a more established antimicrobial background, consistent with its traditional use for the common cold and influenza, scurvy, gastrointestinal and respiratory complaints, vaginitis, headache, and urinary-tract infections [64,65]. In the present study, however, the decisive point is not simply that *R. canina* was active, but that its activity was extraction-selected. Medium-polar fractions, especially WFE/E60, produced the strongest antibacterial profile, whereas *n*-hexane remained consistently weak, indicating that the antibacterial potential of rosehip pseudo-fruits is mainly concentrated in polar-to-amphiphilic phytochemical pools. This agrees with the correlation analysis, where antibacterial ranking aligned primarily with total flavonoids, followed by total phenolics and, more weakly, tannins. Table 9 strengthens this interpretation by showing that WFE/E60 and WFE/ENZ contained flavonols/flavones and related derivatives, including quercetin, kaempferol, luteolin, rutin, catechin, epicatechin, quercetin glycosides, kaempferol derivatives, naringenin/naringenin glycoside, and hesperidin, together with phenolic/hydroxycinnamic acids, organic acids, and tannin/proanthocyanidin-related compounds such as ellagic-acid derivatives and procyanidin-type oligomers. Thus, the antibacterial effect is best interpreted as a flavonoid-weighted, polyphenol-supported phenotype, rather than as a nonspecific effect of crude rosehip extract.

At the mechanistic level, this compound distribution provides a coherent explanation for the observed activity. Flavonoid antibacterial activity is highly structure-dependent, with hydroxylation and prenylation/geranylation often enhancing activity, while some methoxylation patterns may reduce potency [66,67]. Flavonoids may affect bacteria by disturbing membrane and cell-wall integrity, inhibiting nucleic-acid and protein synthesis, interfering with energy metabolism and signal transduction, suppressing efflux pumps, and reducing virulence or biofilm formation [60,68,69,70]. Phenolic and hydroxycinnamic acids may reinforce these effects through membrane permeabilization, intracellular pH disturbance, enzyme inhibition, oxidative stress imbalance, and anti-adhesion activity [61]. Tannin/procyanidin-related structures may add protein-binding, metal-chelating, membrane-interactive, and extracellular-matrix-disrupting effects, which are particularly relevant to antibiofilm activity [63]. Therefore, the stronger activity of the hydroethanolic and enzyme-assisted fractions is likely related to their greater recovery of bioactive compound groups able to affect both bacterial growth and early surface attachment. The available literature on *R. canina* supports an extraction-dependent interpretation of antibacterial activity. Miljković et al. reported MIC values of 4 mg/L against *S. aureus* and 2 mg/mL against *P. aeruginosa* using a hexane: acetone: ethanol extraction system, a solvent mixture with a relatively lipophilic extraction bias that may be less efficient in recovering highly polar antibacterial constituents, including phenolic acids, glycosylated flavonoids, and procyanidin-like compounds [37]. This difference in extraction chemistry may partly explain the lower antibacterial potency reported in that work compared with the stronger activity observed here for hydroethanolic and enzyme-assisted fractions. Montazeri et al. showed solvent-dependent activity, while collection season may partly explain differences from the present September-collected material [32,71]. Other reports using commercial dried pseudo-fruits, 70% ethanol, 96% ethanol, γ-irradiated material, 30% ethanol, aqueous extracts, or methanol extracts likewise produced variable inhibition zones and MICs, ranging from values comparable to ours to substantially weaker activity [72,73,74,75,76]. Overall, the available evidence indicates that the antibacterial activity of *R. canina* is not constant, but depends on the plant matrix and extraction method. In the present study, the strongest fractions were those enriched in the flavonoids, phenolic acids, and tannin/procyanidin-related compounds identified in Table 9.

Biofilms are surface-attached microbial communities encased in a self-produced matrix that enable persistent, device-associated infections [77]. In clinical settings, they promote antimicrobial tolerance via matrix shielding, stress adaptation, and persisted cells, thereby driving relapse and treatment failure [78]. Targeting biofilms formed by *P. aeruginosa*, *K. pneumoniae* and *S. aureus* is clinically pivotal because these pathogens persist on host tissues and indwelling devices as matrix-encased, quorum-sensing, coordinated communities with heightened tolerance to antimicrobials and immune clearance. Plant-derived antibiofilm agents are therefore of particular interest because they can attenuate the earliest stages of biofilm establishment, including initial adhesion, EPS/matrix biogenesis and QS-regulated virulence programmes, without necessarily relying on bactericidal activity. Such anti-virulence, biofilm-disruptive interventions may reduce chronicity and relapse while lowering selective pressure for the emergence of classical antibiotic resistance [78,79].

Across the assay conditions used to induce biofilm development, all *K. pneumoniae*, *P. aeruginosa* and *S. aureus* strains produced measurable surface-associated biomass; however, the magnitude of baseline attachment was markedly strain dependent, with *K. pneumoniae* isolates 94 and 18 at the upper end of the distribution and *P. aeruginosa* 309 consistently at the lower end. Against this biologically heterogeneous starting point, extracts from both plant matrices (CAE and WFE) produced a clear, graded suppression of initial attachment over 0.5 × MIC–4 × MIC, with inhibition becoming the dominant response at ≥MIC, indicating progressive disruption of the earliest colonization steps as exposure increased. Finally, the antibiotic comparators, tested at 0.5×–4× of EUCAST breakpoint-derived MIC values for isolates classified as susceptible, provided a clinically anchored benchmark for this biofilm-initiation (attachment) model, highlighting both the achievable range of antibiofilm suppression with standard-of-care agents and the marked drug–pathogen variability that persists even within susceptible phenotypes. This polarity dependence converges with our phytochemical profiling and supports a polyphenol/flavonoid-centred interpretation of activity. The fractions showing the strongest antibiofilm performance were also those with the highest phenolic/flavonoid loads, particularly WFE/ENZ (TPC 203.34 mg GAE/g; TFC 35.67 mg QE/g), WFE/E60 (TPC 187.00; TFC 34.00), CAE/E60 (TPC 190.00; TFC 27.67), and CAE/ENZ (TPC 160.00; TFC 20.00). Mechanistically, this association is biologically plausible because flavonoids and related phenolics are repeatedly reported to attenuate biofilm establishment by impairing adhesion, perturbing membrane and energy homeostasis, and reprogramming biofilm-regulatory networks, including quorum sensing, thereby reducing maturation and stability even at sub-inhibitory exposures. Supporting precedent exists for flavonols with antibiofilm/anti-virulence activity in the same taxa examined here (e.g., quercetin- and kaempferol-class compounds). Finally, the dominance of polar/enzymatic fractions is strategically significant for *C. autumnale*; given the toxicity liabilities associated with colchicine-class alkaloids, a biofilm-inhibitory signal that tracks with phenolic/flavonoid enrichment provides a defensible basis to prioritize alkaloid-minimized, polyphenol-rich extracts as safer antibiofilm candidates. Consistent with this, QS-focused evidence indicates that phenolics (including flavonoids) can function as quorum-sensing inhibitors in AHL-mediated systems by reducing AHL production, sequestering/inactivating signals, or antagonizing LuxR-type receptors, suppressing QS-regulated phenotypes such as biofilm formation during initiation without requiring bactericidal activity [80].

Studies support the view that *R. canina* antibiofilm activity is strongly contingent on extraction design. Kaya et al. used a microwave-assisted 50% ethanolic rosehip extract and reported 93–100% inhibition of biofilm formation against strong biofilm-forming *S. aureus* and *Listeria monocytogenes*, with MBIC values in the mg/mL range (e.g., 4.4 mg/mL for one *S. aureus* strain); notably, they stress that antibiofilm efficacy varies with both extraction procedure and strain, consistent with our strain-dependent baseline attachment and the superior performance of hydroethanolic/enzyme-assisted fractions [81]. Ivanov et al., 2025, further demonstrated that ethanolic and aqueous *R. canina* fruit extracts can suppress biofilm formation by impairing microbial attachment, detecting inhibition even at sub-inhibitory exposure (0.25 × MIC) and reporting that 0.5 × MIC reduced biofilm biomass to ~46% (ethanolic) and ~54% (aqueous) in a *Candida albicans* model [39]. Although performed in a fungal system, the emphasis on early attachment inhibition at sub-MIC aligns conceptually with our attachment-focused assay and supports interpreting WFE/E40–E60 and WFE/ENZ as fractions enriched in constituents that attenuate biofilm establishment without simply reflecting growth inhibition. From a phytochemical standpoint, the same research group links rosehip antibiofilm activity to a chemically diverse phenolic network—encompassing phenolic acids, ellagic acid derivatives, flavonols, and flavan-3-ols/procyanidins—and explicitly frames the antibiofilm effect as a functional outcome of this polyphenol-rich composition rather than a single dominant constituent [39]. This strengthens a chemistry-based interpretation of our polarity trend: the top-performing fractions are those most expected to concentrate redox-active, adhesion- and matrix-disruptive polyphenols (flavonoids and tannin-like procyanidins); whereas, the weaker *n*-hexane response is consistent with limited partitioning of these polar antibiofilm-relevant pools into non-polar media. Collectively, our results and the rosehip literature converge on a common conclusion: antibiofilm efficacy in *R. canina* is driven primarily by extraction architecture that enriches the phenolic/flavonoid/tannin chemical space, rather than being an invariant property of “rosehip” material.

Regarding our second studied plant, most studies on *C. autumnale* have centred on colchicine-class alkaloids, antioxidant profiling and toxicity, with antibiofilm activity addressed only indirectly or not at all [41,59]. Even where hydroalcoholic flower/organ extracts are chemically characterized for polyphenols, flavonoids and tannins, the literature repeatedly highlights the toxicological liabilities associated with colchicine-rich tissues. Against this background, direct antibiofilm evidence for solvent-partitioned *C. autumnale* flower fractions is essentially absent, positioning our work as a first systematic evaluation of antibiofilm effects during biofilm initiation (attachment) in MDR clinical isolates of *K. pneumoniae*, *P. aeruginosa* and *S. aureus*. In our attachment-focused model, activity was strongly chemistry-dependent: reproducible inhibition concentrated in polar/enzyme-assisted extracts (CAE/E60 and CAE/ENZ), while *n*-hexane was weak/unstable, consistent with polarity-driven enrichment of phenolic pools. Although antibiofilm activity has been demonstrated for colchicine-derived synthetic analogues (including MRSA biofilm inhibition with regulatory effects on ica/agr regulatory systems) [58], the toxicity context of colchicine chemistry reinforces the value of our phenolic-forward, alkaloid-minimizing fractionation strategy for developing safer antibiofilm leads from *C. autumnale* [66].

## 4. Materials and Methods

### 4.1. Plant Materials Collection and Preparation

Two local plant species were used as plant material in this study: (a) *Rosa canina* L. (dog rose), specifically the pseudo-fruits (wild fruits, rose hips; WF); (b) *Colchicum autumnale* L., specifically the large pink to pale lilac flowers (CA). Fresh plant material was col-lected from wild populations in the Epirus region (north-western Greece) during the natural flowering/fruiting period of the species (August–September). Only fully ripe pseudo-fruits of *R. canina* and fully developed flowers of *C. autumnale* were harvested. The qualified taxonomist identified the plant species, and the verified plant materials were submitted for herbarium sheets and voucher specimens. Plant samples were collected by local residents, and voucher specimens for each species were prepared and deposited in the Herbarium of the Laboratory of Botany, Department of Agriculture, University of Ioannina, under assigned reference numbers. The collected plant material was subjected to oven treatment at 65 °C for 12 h to inhibit enzymatic reactions, followed by desiccation in the dark at ambient temperature (23 ± 2 °C) for 7 days [34]. For *C. autumnale*, only petals, calyces and pollen were used, whereas for *R. canina* only the pseudo-fruits (hips) were processed. After complete drying, the samples were ground into a fine powder using a high-speed grinder to obtain a homogeneous material and to facilitate the extraction procedure. The obtained powdered plant material was divided into 5 g aliquots, which were transferred to appropriately labelled vials and stored in the dark at −20 °C until analysis. In each procedure conducted in the present study, the powdered plant materials were first evaluated for microbiological quality; samples exhibiting bacterial or yeast growth were excluded and not used in any subsequent experiments.

### 4.2. Extraction Procedures

**Figure 10:**
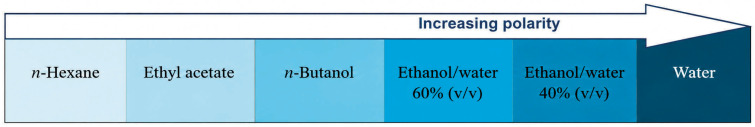
Relative polarity scale of solvents used for plant extraction.

To obtain fractions enriched in bioactive compounds of differing polarity, we combined Soxhlet extraction with maceration and enzymatic hydrolysis, adapting established protocols for the studied plants with minor modifications [82,83,84]. The solvent systems were selected to sequentially recover non-polar, medium-polar and highly polar or enzyme-releasable phytochemicals, in accordance with current recommendations for the preparation of complex natural product matrices (Figure 10).

#### 4.2.1. Soxhlet Extraction (n-Hexane, Ethyl Acetate, n-Butanol)

Powdered *R. canina* pseudo-fruits (WF) and *C. autumnale* flowers (CA) were extracted successively in a Soxhlet apparatus with *n*-hexane, ethyl acetate and n-butanol (solid-to-solvent ratio ~1:10, *w*/*v*) for 5–8 h per solvent. Each extract was dried over anhydrous sodium sulfate, filtered and concentrated under reduced pressure at 40 °C (rotary evaporator). The residue was weighed to calculate extraction yield, transferred to amber vials and stored at 4 °C until analysis The resulting fractions were coded as WF/n-H, WF/EtOAc, WF/n-B and CA/n-H, CA/EtOAc, CA/n-B, respectively. In each code, the first part denotes the plant material (WF: *Rosa canina* pseudo-fruits, “wild fruits”; CA: *Colchicum autumnale* flowers) and the second part denotes the extraction solvent (n-H, *n*-hexane, EtOAc: ethyl acetate, n-B: n-butanol).

#### 4.2.2. Maceration Extracts (Aqueous and Hydroethanolic; A, E40, E60)

For maceration, plant powders (WF or CA) were mixed, with solvent at a ratio of 1:2 (*w*/*v*) in sterile Erlenmeyer flasks and agitated at 30 °C for 24 h. Three solvent systems were used: distilled water (aqueous extract, A) and hydroethanolic mixtures of 40% and 60% (*v*/*v*) ethanol (E40 and E60, prepared from 96% ethanol with distilled water). After extraction, suspensions were filtered (Whatman No. 1), deep-frozen at −80 °C and concentrated under reduced pressure at 40 °C. The concentrates were lyophilised, weighed and stored at −20 °C in the dark. Immediately before use, dry extracts were exposed to UV light overnight to minimize microbial contamination.

#### 4.2.3. Enzymatic Extract (ENZ)

An enzymatically hydrolysed extract was prepared to release enzyme-accessible low-molecular-weight bioactive compounds with possible antibacterial activity, following a pepsin-based protocol with minor modifications [83,84,85]. Fresh *R. canina* pseudo-fruits (WF) or *C. autumnale* flowers (CA) were first washed under running water, manually cleaned to remove unsuitable parts and cut into ~1 mm pieces using a slicer to increase the available surface area. The cut material was then rinsed with phosphate-buffered saline (PBS) to remove intracellular material released from damaged cells. Pre-treated plant material was immersed in distilled water at a plant-to-water ratio of 1:2 (*w*/*w*) and the suspension was acidified to pH 2.0 with concentrated HCl. Pepsin (P7000/P7125, 250–400 units/mg, Merck KGaA, Darmstadt, Germany) was added to a final concentration of approximately 1.0% (*w*/*w*) relative to the plant material. The mixture was incubated at 37 °C for 48 h under gentle agitation. Enzymatic hydrolysis was terminated by heating the suspension at 80–90 °C for 10 min. The resulting slurry was divided into ~100 g portions, manually pressed with a sterile pestle and filtered through sterilized Whatman No. 1 filter paper. Filtrates were concentrated under reduced pressure using a rotary evaporator (KNF RC 900; KNF Neuberger GmbH, Breisgau, Germany), deep-frozen at −80 °C and lyophilised. The resulting powders were designated WF/ENZ and CA/ENZ (collectively referred to as ENZs) and stored at −20 °C until further use.

#### 4.2.4. Extract Abbreviations and Preparation of Stock Solutions

For clarity and comparability, the following abbreviations were assigned to the *R. canina* (WF) and *C. autumnale* (CA) extracts:

WF extracts (*R. canina* pseudo-fruits): WF/*n*-Hexane (WFE/n-H); WFE/ethyl acetate (WFE/EtOAc); WF/n-butanol (WFE/n-B); WF/aqueous (WFE/A); WF/ethanolic 40% (WFE/E40); WFE/ethanolic 60% (WFE/E60); WF/enzymatic (WFE/ENZ). CA extracts (*C. autumnale* flowers): CA/*n*-Hexane (CAE/n-H); CA/ethyl acetate (CAE/EtOAc); CAE/n-butanol (CAE/n-B); CA/aqueous (CAE/A); CA/ethanolic 40% (CAE/E40); CA/ethanolic 60% (CAE/E60); CA/enzymatic (CAE/ENZ).

All crude extracts were protected from direct light and stored at low temperature to minimize photodegradation and oxidative degradation of labile constituents. For biological and phytochemical assays, stock solutions were prepared immediately before use. The required mass of each crude extract was accurately weighed and dissolved in 2% (*v*/*v*) aqueous dimethyl sulfoxide (DMSO) to obtain a final stock concentration of 10 mg/mL. Preliminary tests confirmed that 2% DMSO did not exert detectable antibacterial effects on the tested strains and was therefore considered an appropriate, non-toxic solvent control. Lyophilized aqueous crude extracts (WF/A and CA/A) were reconstituted in a mixed aqueous solvent composed of boiled water and 95% methanol in a 1:1 (*v*/*v*) ratio to improve solubility while maintaining compatibility with microbiological and spectrophotometric assays. All solutions were prepared under sterile conditions, and all experiments (microbiological, antioxidant and biophysical assays) were performed in triplicate.

### 4.3. Phenotypic Profiling of Bioactive Molecules in Botanical Fractions

All extracts obtained in this study were subjected to qualitative (“phenotypic”) screening for major classes of secondary metabolites, including alkaloids, anthraquinones, terpenoids, steroids, saponins, tannins, cardiac glycosides, total phenolics, flavonoids, carotenoids and ascorbic acid. Classical colour and precipitation reactions were performed according to established protocols and subsequent methodological updates, with minor adaptations for the matrices under study. Briefly, 1–2 mL of each extract (10 mg/mL) was used per assay, with the corresponding solvent serving as a negative control. The outcome of each reaction (characteristic colour change or precipitate formation) was recorded as present (+) or absent (−) and is summarized for all extracts in Table 1, in the Results section, while the individual tests—including target class, reagents and expected visual endpoints—are detailed in Table 10.

#### 4.3.1. Determination of Total Phenolic Content

Total phenolic content was quantified by a Folin–Ciocalteu colorimetric assay adapted to a microplate format. Aliquots of each extract stock were diluted in methanol to obtain working solutions between 4 and 40 µg/mL. For each determination, 15 µL of the appropriately diluted sample was dispensed into a well of a 96-well plate and combined with 120 µL of Folin–Ciocalteu reagent. After a reaction period of 5 min at room temperature, 120 µL of Na_2_CO_3_ solution (60 g/L) was added to initiate chromophore development. The plates were gently agitated and left to react for 90 min in the dark to ensure full colour stabilization. The absorbance of each well was then read at 725 nm using a microplate spectrophotometer, with solvent and reagent blanks processed in parallel. Quantification was performed by interpolation on a calibration curve constructed with gallic acid standards (2–40 µg/mL in methanol), and results were expressed as milligrams of gallic acid equivalents per gram of dry extract (mg GAE/g) [100].

#### 4.3.2. Determination of Total Flavonoid Content

Total flavonoid content was determined by an AlCl_3_/NaNO_2_ colorimetric assay. Extracts were dissolved in methanol (1–2 mg/mL), and 0.50 mL of each solution was transferred to a 10 mL volumetric flask containing 4.0 mL deionized water. Then 0.30 mL of 5% (*w*/*v*) NaNO_2_ was added and the mixture was left for 5 min at room temperature. Subsequently, 0.30 mL of 10% (*w*/*v*) AlCl_3_ was added; at the 6th minute, 2.0 mL of 1 M NaOH was introduced and the volume was adjusted to 10 mL with water. Formation of flavonoid–Al^3+^ complexes yielded a stable orange–yellow chromophore, and absorbance was measured at 510 nm after 10 min against reagent blanks. Quantification was based on an external calibration curve constructed with rutin standards in methanol, and results were expressed as milligrams of rutin equivalents per gram of dry extract (mg RE/g) [100].

#### 4.3.3. Determination of Total Tannin Content (TTC)

Total tannin content was quantified by a protein-precipitation/Ferric chloride colorimetric assay adapted from Hagerman and Butler, which exploits the strong affinity of tannins for proteins and their subsequent reaction with Fe^3+^ in alkaline medium. Briefly, an aliquot of each sample was dissolved in water to obtain a working concentration of 400 µg/mL. One millilitre of this solution was mixed with 2 mL of bovine serum albumin (BSA, 1 mg/mL in 0.4 M acetate buffer, pH 6.0) and incubated for 24 h at 4 °C in the dark to allow selective precipitation of protein–tannin complexes. The mixtures were then centrifuged (900× *g*, 15 min, 4 °C) and the supernatant discarded. The resulting pellets were re-dissolved in 4 mL of sodium dodecyl sulfate/triethanolamine solution (SDS/TEA, 1%/5%, *w*/*v*), after which 1 mL of FeCl_3_ (0.01 M in 0.01 M HCl) was added. Following a 15 min incubation in the dark, the absorbance of the chromogenic iron–tannin complexes was measured at 510 nm against a reagent blank containing SDS/TEA and FeCl_3_ but no sample. Quantification was performed using an external calibration curve constructed with tannic acid standards (10–190 µg/mL), and total tannin content was expressed as milligrams of tannic acid equivalents per gram of dry extract (mg TAE/g) [101].

### 4.4. In Vitro Studies Antioxidant Assays

#### 4.4.1. DPPH Radical-Scavenging Assay

The DPPH assay was used as a primary measure of radical-scavenging capacity. This method relies on the reduction in the stable nitrogen-centred radical 2,2-diphenyl-1-picrylhydrazyl (DPPH), which exhibits a strong absorbance at 517 nm and is bleached upon reaction with hydrogen- or electron-donating antioxidants. A freshly prepared methanolic DPPH solution (final concentration 200 µM in the wells) was dispensed into 96-well plates and mixed 1:1 (*v*/*v*) with serial dilutions of each extract in methanol (0.1–500 µg/mL). Methanol plus DPPH served as the negative control, and gallic acid (0.1–5 µg/mL) was used as a reference antioxidant. After gentle mixing, plates were incubated for 30 min at 25 ± 2 °C in the dark, and residual DPPH was quantified at 517 nm using a microplate reader.

The scavenging activity was expressed as the percentage (%) of DPPH inhibition and was calculated for each concentration using the following equation:

$$\mathrm{Scavenging}\;\mathrm{effect},\;\mathrm{as}\;\mathrm{DPPH}\;\mathrm{inhibition}\;(\%)\;=\;[A_{\mathrm{control}}-\;A_{\mathrm{sample}}/A_{\mathrm{control}}]\;\times \;100$$

where $A_{\mathrm{control}}$ and $A_{\mathrm{sample}}$ are the absorbances of the DPPH solution without and with extract, respectively. Dose–response curves (% inhibition versus concentration) were constructed, and the IC_50_ value (µg/mL of extract required to reduce the initial DPPH absorbance by 50%) was obtained by regression analysis. All measurements were performed in triplicate.

#### 4.4.2. Reducing Power Assay (Of Fe^3+^ to Fe^2+^ System)

The electron-donating capacity of the extracts was evaluated by a potassium ferricyanide–ferric chloride reducing power assay, which monitors the reduction in Fe^3+^ to Fe^2+^ as an index of phenolic-based redox activity. Increasing absorbance at 700 nm reflects higher reducing power [102]. Briefly, graded amounts of each extract (25–250 μg) were dissolved in methanol (1.0 mL), mixed with 1.0 mL of phosphate buffer (0.2 M, pH 6.6) and 1.0 mL of 1% (*w*/*v*) potassium ferricyanide. The mixtures were incubated at 50 °C for 20 min, after which 1.0 mL of 10% (*w*/*v*) trichloroacetic acid was added to stop the reaction. Samples were centrifuged at 3000 rpm for 10 min, and 2.0 mL of the supernatant was combined with 2.0 mL distilled water and 0.4 mL of 0.1% (*w*/*v*) ferric chloride. After 10 min at room temperature, the formation of the Fe^2+^–ferricyanide complex (Prussian blue) was quantified by measuring absorbance at 700 nm against a reagent blank. All measurements were performed in triplicate, and higher absorbance values were interpreted as greater reducing power. All determinations were performed in triplicate.

### 4.5. Antibacterial Activity and Biofilm Modulation Against Clinical Isolates

#### 4.5.1. Bacterial Strains and Antimicrobial Susceptibility Testing

The antibacterial activity of the extracts was evaluated against eight multidrug-resistant clinical isolates and three reference strains. The clinical panel comprised five *K. pneumoniae* bloodstream isolates, two *P. aeruginosa* isolates (one from blood, one from bronchoalveolar lavage), and one *S. aureus* bloodstream isolate. In addition, the following quality-control strains were included: *P. aeruginosa* ATCC 27853, *K. quasipneumoniae ATCC 700603* and *S. aureus* ATCC 25923. All clinical isolates were identified to species level using a Microflex LT™ MALDI-TOF MS instrument (Bruker Daltonik GmbH, Bremen, Germany). For the Gram-negative isolates, the nucleotide sequence of the entire genome of the clinical strains was determined [103,104] and the resulting sequences were analyzed with the ResFinder v4.1 software (https://cge.food.dtu.dk/services/ResFinder/, accessed on 22 December 2025). Specifically, ResFinder was used to search for antibacterial resistance genes and their copies in each strain, as well as other mechanisms that may contribute to resistance to newer antibacterial drugs, such as efflux pumps and loss of porins. The strains originated from distinct patients hospitalized in tertiary-care hospitals and were obtained from the Bacterial Collection of the Department of Microbiology, Medical School, National and Kapodistrian University of Athens.

Phenotypic antimicrobial susceptibility testing and determination of minimum inhibitory concentrations (MICs) for conventional antibiotics were performed using the VITEK 2 automated system and Etest gradient diffusion strips (bioMérieux, Marcy l’Etoile, France), in accordance with the manufacturer’s instructions and interpreted according to current EUCAST clinical breakpoints.

#### 4.5.2. Preliminary Antibacterial Screening by Agar-Based Disc Diffusion Assay

The antibacterial activity of the extracts was assessed by a disc diffusion assay adapted from CLSI guidelines (2015) with minor modifications [105]. Clinical isolates *of K. pneumoniae* and *P. aeruginosa* were grown overnight in Luria–Bertani broth, and *S. aureus* on 5% sheep blood Columbia agar, at 37 °C for 18–24 h. From the fresh cultures, a single colony was suspended in sterile 0.85% saline and adjusted to 0.5 McFarland (≈10^8^ CFU/mL) using a turbidimeter. This standardized inoculum was immediately spread onto dried Mueller–Hinton agar plates to obtain a uniform lawn. Sterile 6 mm Whatman No. 1 paper discs were loaded with each plant extract at 10%, 20%, 50% and 100% (*v*/*v*), prepared in 2% (*v*/*v*) aqueous dimethyl sulfoxide (DMSO; Honeywell, Charlotte, NC, USA), or in sterile distilled water for aqueous extracts. For each loading, discs were impregnated under identical conditions and allowed to drain briefly to remove excess liquid prior to air-drying for ~20 min, to minimize variability in disc content. Discs were then placed onto agar plates previously inoculated with the test organism. Discs containing 2% DMSO or sterile distilled water served as negative controls, while commercial antibiotic discs were included as positive controls. Plates were incubated at 37 °C for 18–24 h, after which inhibition zones were photographed and quantified using ImageJ ImageJ software version 1.54. For each condition, zone diameters were measured along multiple axes and averaged. All assays were performed in three independent replicates.

#### 4.5.3. Determination of Minimum Inhibitory Concentration (MIC) and Minimum Bactericidal Concentration (MBC)

MIC and MBC values for the extracts were determined by a broth microdilution assay in 96-well microplates, adapted from standard methods for plant products [83,84]. Crude extracts were first dissolved at 400 mg/mL in 2% (*v*/*v*) aqueous dimethyl sulfoxide (DMSO), a concentration previously verified as non-inhibitory for the tested bacteria; lyophilised aqueous extracts were reconstituted in ultrapure water. Two-fold serial dilutions were then prepared in sterile medium to obtain a final concentration range of approximately 200 to 0.0125 mg/L in the assay wells. Bacterial inocula were prepared from fresh overnight cultures and adjusted spectrophotometrically to ~5 × 10^5^ CFU/mL in cation-adjusted Mueller–Hinton broth. Sterility controls (medium only), growth controls (medium plus inoculum) and solvent controls (medium + 2% DMSO + inoculum) were included on every plate. Microplates were incubated at 37 °C for 24 h. Following incubation, bacterial growth was assessed visually and by addition of an aqueous solution of triphenyltetrazolium chloride (TTC, 5 g/L; Sigma-Aldrich, St. Louis, MO, USA), which is reduced to a red formazan product by metabolically active cells. The MIC was defined as the lowest extract concentration showing neither visible turbidity nor TTC colour development compared with the growth control. To determine the MBC, 20 µL aliquots from all wells at and above the MIC (i.e., wells without visible growth) were spot-inoculated onto Mueller–Hinton agar plates and incubated at 37 °C for up to 24 h. Plates were examined for colony formation, and the MBC was defined as the lowest extract concentration resulting in complete absence of growth, corresponding to ≥99.9% reduction in the initial inoculum. All MIC and MBC determinations were performed in triplicate.

#### 4.5.4. Initial Attachment Inhibition (Biofilm Initiation) Assay

To assess inhibition of initial cell attachment and biofilm initiation, biofilm formation was quantified in 96-well flat-bottom polystyrene microplates using a crystal-violet staining protocol [106,107]. Each well received 100 µL of a standardized bacterial suspension (≈10^8^ CFU/mL), 100 µL of Mueller–Hinton (MH) broth and 100 µL of the test extract at the selected sub-MIC. Plates were incubated aerobically at 37 °C for 24 h to allow surface colonization in the continuous presence of the extract. This design evaluates prevention of biofilm establishment during the attachment/formation phase and does not test disruption or eradication of pre-established (mature) biofilms. In parallel, the intrinsic biofilm-forming capacity of all isolates was confirmed in the absence of extracts, using media optimized for each species: double-strength Luria–Bertani broth with 0.1% glucose for *P. aeruginosa*, tryptic soy broth with 1% (*w*/*v*) glucose (TSBG) for *S. aureus*, and brain–heart infusion (BHI) broth supplemented with 2% (*w*/*v*) glucose for *K. pneumoniae*. For each strain, 100 µL of inoculum adjusted to ≈10^8^ CFU/mL was dispensed per well and incubated at 37 °C for 24–36 h. After incubation (with or without extract), planktonic cells were gently removed, and wells were washed twice with pre-warmed PBS to eliminate non-adherent bacteria. The remaining attached biomass was fixed with 200 µL of absolute methanol for 15 min, air-dried, and stained with 200 µL of 0.4% (*w*/*v*) crystal violet for 5 min. Excess dye was rinsed off under a gentle stream of tap water, and plates were allowed to dry completely. The bound stain was then solubilized with 160 µL of 33% (*v*/*v*) glacial acetic acid per well, thoroughly mixed, and 100 µL of the resulting suspension was transferred to a fresh microplate. Optical density (OD) was measured at 630 nm. Antibiofilm activity was expressed as the percentage reduction in biofilm biomass relative to the untreated control, after correction for the DMSO solvent control, according to:

Biofilm Inhibition% = [(OD Positive Control − OD Experimental)/(OD Positive  Control)] × 100

Based on the results, the tested plant extracts were classified as: Excellent (+++++) AB activity (>95% inhibition); Very Good (++++) AB activity (95–85% inhibition); Good (+++) AB activity (85–70% inhibition); Moderate (++) AB activity (50–70% inhibition); Poor (+) AB activity (more than 0–50% inhibition); No (-) AB activity (0% or less). AB meaning: antibiofilm activity.

### 4.6. UHPLC-DAD-MS/MS Profiling of the Most Bioactive Extracts

To obtain a more resolved phytochemical annotation of the most relevant fractions, selected extracts were further analyzed by UHPLC-DAD-MS/MS. Based on the initial phytochemical and bioactivity screening, CAE/E60, CAE/ENZ, WFE/E60, and WFE/ENZ were selected for targeted chromatographic profiling, as these extracts provided the best overall balance of phytochemical enrichment, antioxidant capacity, antibacterial activity, and inhibition of early biofilm attachment among the tested fractions. Separation was performed on a C18 reversed-phase column (100 × 2.1 mm, 2.6 μm) at 40 °C using water with 0.1% formic acid (A) and acetonitrile with 0.1% formic acid (B) at a flow rate of 0.40 mL/min under gradient elution. DAD data were acquired at 245, 280, 320, and 360 nm, and MS/MS detection was performed in positive and negative electrospray ionization modes over *m*/*z* 100–1000 using stepped collision energy. Extracts were reconstituted in methanol:water (50:50, *v*/*v*) containing 0.1% formic acid, sonicated, centrifuged, filtered through 0.22 μm membranes, and analyzed under the above conditions. Compound annotation was based on retention time, UV spectra, precursor ions, and MS/MS fragmentation patterns, supported by reference standards where available and literature comparison. Only compounds consistently detected in all three replicate injections with identification confidence >90% were retained in the final annotation tables.

### 4.7. Statistical Analysis

Results are presented as the mean of three independent replicates ± the corresponding standard deviation. Differences among groups (TPC/TFC/TTC of the extracts/fractions; antioxidant activity by DPPH and FRAP assays; disc-diffusion inhibition-zone diameters; and MIC and MBC values across the tested pathogens and treatments) were evaluated using one-way analysis of variance (ANOVA), followed by Tukey’s HSD post hoc test for multiple comparisons. Prior to ANOVA, the Shapiro–Wilk test was applied to verify normality of residuals. When normality was not satisfied, comparisons among groups were performed using the Kruskal–Wallis test with appropriate post hoc multiple comparisons. Kendall’s tau-c coefficient was used for correlations between the contents of the basic phytochemicals and the antibacterial effects. All statistical analyses were performed using SPSS v28 (IBM Corp., Armonk, NY, USA).

## 5. Limitations and Future Directions

In the present study, flowers of *C. autumnale* and pseudo-fruits of *R. canina* were evaluated for antioxidant capacity and antibacterial/antibiofilm activity using solvent-partitioned fractions obtained under multiple extraction conditions. *C. autumnale* is traditionally associated with toxicity and anti-inflammatory use, and modern pharmacology has linked colchicine-class alkaloids from *Colchicum* tissues to clinically relevant bioactivities, including antimitotic and antitumor effects. To our knowledge, this is the first study to systematically assess the antibacterial and antibiofilm potential of *C. autumnale* flower-derived fractions against a diverse collection of bacterial strains, including multidrug-resistant (MDR) clinical isolates. *R. canina* pseudo-fruits have previously been investigated for antibacterial/antibiofilm effects; however, most studies have used fewer extraction systems and more limited strain panels. In this work, the comparative design across multiple extraction conditions and MDR isolates expands the available evidence base. Although measurable differences were observed among fractions across antioxidant, antibacterial, and early biofilm-initiation assays, several limitations should be considered when interpreting the findings and defining the next research steps.

A key limitation of the present study is that the biological evaluation was performed mainly on crude, multi-component extracts, whose composition was shaped by solvent polarity and extraction design. This limitation is partially addressed by the compound-level phytochemical profiling of the most active fractions presented in Table 9. Specifically, CAE/E60, CAE/ENZ, WFE/E60, and WFE/ENZ were selected for further characterization because they showed the most relevant combined antioxidant, antibacterial, and antibiofilm performance. Their profiling confirmed the presence of chemically diverse bioactive pools, including flavonoids, phenolic acids, hydroxycinnamic acids, catechin/tannin-related compounds, organic acids, and, in *C. autumnale*, colchicine-related tropolone alkaloids. However, these data should be interpreted as supportive chemical evidence, not as definitive proof of compound-specific activity. The profiling strengthens the link between extraction strategy, phytochemical enrichment, and biological performance, but it does not identify which individual compounds are responsible for the observed effects, nor whether the activity results from additive, synergistic, or antagonistic interactions within each extract. This distinction is particularly important for *C. autumnale*, where colchicine-class alkaloids are both biologically relevant and toxicologically sensitive. Therefore, further quantitative LC–MS/MS analysis, isolation of candidate constituents, cytotoxicity assessment, and interaction/synergy testing are required to define active principles, evaluate safety, and support marker-based standardization. Overall, the present work should be regarded as a microbiology-driven screening and prioritization study, strengthened by targeted phytochemical profiling of the leading fractions, rather than as a complete chemical, pharmacological, and toxicological characterization of the full extract panel.

Moreover, disc diffusion outcomes obtained with crude plant extracts should be interpreted with caution, because inhibition-zone diameters integrate true antibacterial effects with matrix-dependent diffusion kinetics in agar. Diffusion is strongly governed by physicochemical properties (e.g., polarity, molecular size, ionization state, viscosity, and interactions among constituents) and by the undefined mass and composition of complex mixtures. Consequently, inhibition zones from crude fractions are not directly comparable to CLSI/EUCAST breakpoint frameworks, which are calibrated for standardized antibiotic discs with known drug loads, diffusion characteristics, and interpretive criteria. Accordingly, disc diffusion was used here primarily as a comparative screening readout, whereas MIC/MBC determinations provide the primary concentration-based assessment of antibacterial activity.

Importantly, given the known toxicological liabilities of *C. autumnale*, particularly its colchicine-class chemistry, the absence of cytotoxicity/biocompatibility testing represents a major constraint on interpretation and on any discussion of practical applicability. Establishing whether antibacterial activity and inhibition of early biofilm initiation can be retained in fractions with an improved safety profile is essential before progressing beyond in vitro screening. In this context, the observation of measurable effects across multiple MDR clinical isolates provides a strong rationale for next-stage work integrating (i) targeted chromatographic profiling and marker-based standardization of the most active fractions and (ii) cytotoxicity-guided fractionation to define activity windows with improved safety margins.

Future studies should also determine whether selected fractions enhance the activity of conventional antibiotics against MDR pathogens when used in combination. This is a logical extension of the present findings because complex botanical fractions contain multiple constituents that may function as antibiotic adjuvants, for example by increasing membrane permeability, inhibiting efflux systems, modulating redox homeostasis, or altering bacterial stress-response and biofilm-initiation pathways. Such mechanisms may shift the apparent susceptibility phenotype even when direct antibacterial potency is moderate. Combination effects should be evaluated using established quantitative approaches (e.g., checkerboard assays with fractional inhibitory concentration indices, supported by time–kill kinetics and, where appropriate, biofilm-initiation/early-attachment combination assays). Importantly, interaction outcomes should be interpreted alongside targeted chromatographic profiling and cytotoxicity/biocompatibility assessment, so that potentiating effects can be attributed to chemically characterized fractions and prioritized within defined, safety-relevant activity windows.

## 6. Conclusions

The conclusions presented here are constrained by the study design and should be interpreted within the methodological and experimental limitations described above.

- Organs (flowers of *C. autumnale* and pseudo-fruits of *R. canina*) were extracted using seven extraction systems spanning a polarity gradient (including enzyme-assisted processing), yielding fractions with substantial levels of total phenolics, total flavonoids, and tannins. Across the study endpoints, solvent polarity (and enzymatic assistance) consistently shaped the qualitative/quantitative phytochemical profiles and, in turn, the measured bioactivities.
- For *C. autumnale*, n-butanol, 60% ethanol, and enzymatic fractions recovered the highest levels of the measured phytochemical pools, whereas for *R. canina* the highest recoveries were generally associated with enzymatic, 40% ethanol, and n-butanol systems—supporting these extraction conditions as the most efficient routes to phenolic-/flavonoid-enriched fractions in the matrices studied.
- In the DPPH assay, the most pronounced radical-scavenging activity for *C. autumnale* was observed for n-butanol, ethyl acetate, and 60% ethanol fractions, while for *R. canina* the strongest performance was associated with enzymatic and hydroethanolic (40–60%) fractions. Overall, antioxidant performance aligned with phenolic-/flavonoid-enriched fractions rather than non-polar fractions.
- In the FRAP assay (as FRAP absorbance), *C. autumnale* fractions generally showed lower reducing power than the reference antioxidants, although n-butanol, ethyl acetate, and enzymatic fractions exhibited comparatively higher reducing capacity within the *C. autumnale* extract set. For *R. canina*, the enzymatic fraction displayed particularly strong FRAP performance (approaching the reference antioxidants under these assay conditions), followed by 60% ethanol and ethyl acetate fractions.
- Extracts from both plants produced measurable growth inhibition of clinical isolates and reference strains in vitro, with the largest inhibition zones typically observed for 60% ethanol and n-butanol fractions for *C. autumnale* and for 60% ethanol and enzymatic fractions for *R. canina*. MIC/MBC determinations further supported a fraction-dependent ranking of antibacterial activity, indicating that activity was concentrated primarily in medium-polar and enzyme-assisted fractions.
- In the initial-attachment/early biofilm model against major biofilm-forming pathogens (*K. pneumoniae*, *P. aeruginosa*, and *S. aureus*), 60% ethanol, ethyl acetate, and enzymatic fractions from both plant matrices produced the most pronounced reductions in early biofilm establishment under the tested conditions, extending the activity profile beyond planktonic growth endpoints.
- Taken together, these results support the view that extraction architecture is a dominant driver of phytochemical enrichment and of the antioxidant and antibacterial phenotypes observed in vitro, and they identify specific polarity windows (medium-polar/polar and enzyme-assisted systems) as the most informative fractions for deeper chemical and biological resolution in subsequent work.

## Figures and Tables

**Table 1:** Total phenolic, flavonoid and tannin contents of *Colchicum autumnale* flower and *Rosa canina* pseudo-fruit extracts of solvent-partitioned fractions (mean ± SD, n = 3) with Kruskal–Wallis *p*-values and post hoc lettering. Notes: For each compound (row) within a plant source, Kruskal–Wallis *p*-values test differences among solvent fractions. Within each row, different superscript letters denote significant pairwise differences (*p* < 0.05) among extracts using a Tukey-type multiple-comparisons procedure applied to rank-transformed values; entries sharing at least one letter are not significantly different.

No	Plant Extract /Solvent	*Colchicum autumnale*’s Extracts by Different Solvents			*Rosa canina*’s Extracts by Different Solvents		
		Total Phenolics (in mg Gallic Acid Equivalent)/g of Dry Weight of the Extract (*p* = 0.003538)	Total Flavonoids (in mg Quercetin Equivalents) per g of Dry Weight of the Extract (*p* = 0.008675)	Total Tannins (in mg Tannic Acid Equivalent)/g Dry Weight of the Extract (*p* = 0.00475)	Total Phenolics (in mg Gallic Acid Equivalent)/g of Dry Weight of the Extract (*p* = 0.003763)	Total Flavonoids (in mg Quercetin Equivalents) per g of Dry Weight of the Extract (*p* = 0.004195)	Total Tannins (in mg Tannic Acid Equivalent)/g Dry Weight of the Extract (*p* = 0.003612)
1	*n*-Hexane	5 ± 1 ^d^	5.34 ± 0.58 ^b^	1 ± 0.2 ^c^	33.67 ± 3.21 ^d^	2.5 ± 0.5 ^d^	2.00 ± 0.2 ^d^
2	Ethyl acetate	37 ± 1.73 ^b^	2.3 ± 1 ^a^	12.67 ± 0.58 ^b^	53.00 ± 2.65 ^cd^	13.67 ± 1.53 ^c^	11.00 ± 1.00 ^d^
3	n-Butanol	50.33 ± 2.52 ^a^	24.67 ± 4.04 ^a^	20 ± 1 ^a^	110.00 ± 17.80 ^c^	20.67 ± 3.06 ^b^	31.34 ± 3.06 ^c^
4	Aqueous	14.34 ± 3.06 ^d^	5 ± 1.73 ^b^	7 ± 1 ^c^	151.33 ± 2.31 ^b^	13.00 ± 1.73 ^cd^	46.34 ± 1.53 ^ab^
5	Ethanol 40%	22.33 ± 0.58 ^c^	12.67 ± 2.52 ^b^	12.33 ± 0.58 ^b^	190.00 ± 10.00 ^a^	27.67 ± 2.52 ^a^	40.00 ± 2.00 ^b^
6	Ethanol 60%	35.67 ± 2.52 ^b^	20 ± 2 ^a^	13 ± 1 ^b^	125.00 ± 5.00 ^b^	16.00 ± 1.73 ^bc^	33.00 ± 1.00 ^c^
7	Enzymatic	29.34 ± 2.31 ^c^	21.33 ± 3.06 ^a^	17.33 ± 0.58 ^a^	203.34 ± 11.55 ^a^	35.67 ± 3.06 ^a^	53.00 ± 2.65 ^a^

**Table 2:** DPPH Radical-Scavenging potency (IC_50_, μg/mL) of solvent-partitioned fractions of plants extracts. Notes: IC_50_ values were estimated by linear interpolation of replicate (% inhibition vs. concentration) curves (values are the mean of three replicates). Fractions not reaching 50% inhibition at 500 μg/mL are reported as >500 μg/mL. Within each plant column, different superscript letters indicate significant differences among fractions (Kruskal–Wallis: CA *p* = 0.003147; WF *p* = 0.003331; post hoc Dunn’s test with Holm adjustment, *p* < 0.05).

Solvent/Fraction	CA IC_50_ (μg/mL)	WF IC_50_ (μg/mL)
*n*-Hexane	>500 ᵇ	>500 ᵇ
Ethyl acetate	66.26 ± 0.77 ᵃ	44.96 ± 1.63 ᵃ
n-Butanol	57.83 ± 1.10 ᵃ	47.71 ± 1.39 ᵃ
Aqueous	333.75 ± 6.44 ᵃ	40.43 ± 0.30 ᵃ
Ethanol 40%	80.20 ± 0.84 ᵃ	37.41 ± 0.33 ᵃ
Ethanol 60%	55.06 ± 1.28 ᵃ	39.55 ± 0.31 ᵃ
Enzymatic	38.47 ± 1.37ᵃ	33.60 ± 0.45 ᵃ

**Table 3:** FRAP reducing power of solvent-partitioned fractions from *C. autumnale* flowers (CA) and *R. canina* pseudo-fruits (WF), expressed as EC_50_ (μg/mL). Notes: Values are expressed as mean ± SD (n = 3 independent experiments). EC_50_ values (μg/mL) were estimated by linear interpolation of replicate (absorbance at 700 nm vs. concentration) curves; EC_50_ was defined as the concentration required to reach 50% of the response range (midpoint between minimum and maximum absorbance) for each replicate curve. Within each plant column, different superscript letters indicate significant differences among fractions (Kruskal–Wallis: CA *p* = 0.0510; WF *p* = 0.00773; post hoc Dunn’s test with Holm adjustment, *p* < 0.05).

Solvent/Fraction	CA EC_50_ (μg/mL)	WF EC_50_ (μg/mL)
*n*-Hexane	140.96 ± 27.65 ᵃ	126.94 ± 7.28 ᵇ
Ethyl acetate (EtOAc)	106.15 ± 0.93 ᵃ	104.92 ± 1.20 ᵃ
n-Butanol (n-BuOH)	151.65 ± 44.73 ᵃ	102.79 ± 1.74 ᵃ
Aqueous	130.17 ± 1.42 ᵃ	105.94 ± 1.25 ᵃ
Ethanol 40% (E40)	116.44 ± 0.93 ᵃ	100.07 ± 0.61 ᵃ
Ethanol 60% (E60)	121.32 ± 0.87 ᵃ	100.72 ± 0.74 ᵃ
Enzymatic (ENZ)	117.82 ± 1.75 ᵃ	100.50 ± 0.92 ᵃ

**Table 4:** Phenotypic antimicrobial susceptibility of the studied *S. aureus* isolate and reference strains against common antibiotic agents (Interpretation values according to the EUCAST guidelines.

Bacterial Strains			Antimicrobial Agents MIC Values mg/L														
ID	Isolate	Isolate Source	PEN/G	CEF screen	OXA	VAN	TMP-SMX	ERY	CLI	GEN	MFX	CIP	Q/D	LIN	DAP	TEC	TIG
78	*S. aureus*	Blood	**>0.25**	**Positive**	**2**	**<=0.5**	**<=10**	**<=0.25**	**<=0.25**	**8**	**<=0.25**	**<=5**	**<=0.25**	**1**	**1**	**1**	**<=0.12**
Ref.	*S. aureus*, ATCC 25923	ATTC	**<0.25**	**Negative**	**<=0.25**	**1**	**2**	**<=0.25**	**<=0.25**	**0.25**	**<=0.25**	**0.25**	**<=0.25**	**2**	**1**	**1**	**<=0.12**

**Abbreviations**: PEN/G, Benzylpenicillin; CEF Screen, Cefoxitin screen; OXA, Oxacillin; VAN, Vancomycin; TMP-SMX, Trimethoprim/Sulfamethoxazole; ERY, Erythromycin; CLI, Clindamycin; GEN, Gentamicin; MFX, Moxifloxacin; CIP, Ciprofloxacin; Q/D, Quinupristin/Dalfopristin; LIN, Linezolid; DAP, Daptomycin; TEC, Teicoplanin; TIG, Tigecycline; Inducible clindamycin resistance: NEG (-). Colour interpretation: Red: resistant (R); Blue: susceptible (S).

**Table 5:** Ranking distribution of inhibition-zone diameters for *C. autumnale* flower-derived extract preparations across all strain × disc-content conditions (n = 44 per extract; based on Supplementary Material File S3). Aqueous and n-hexane extracts were excluded because they did not yield large inhibition zones. Overall distribution differed significantly from uniformity (χ^2^ = 58.8663, *p* < 0.001).

Extracts’ Preparation	Largest	2nd Largest	3d Largest	Top-3 (n, %)	Other	Total
Ethanol 40%	-	1	5	6 (13.6)	38	44
Ethanol 60%	24	17	2	43 (97.7)	1	44
Enzymatic	11	9	12	32 (72.7)	12	44
Ethyl acetate	-	1	10	11 (25.0)	33	44
n-Butanol	9	16	15	40 (90.9)	4	44
Total	44	44	44	100	88	220

**Table 6:** Ranking distribution of inhibition-zone diameters for *R. canina* pseudo-fruit extract preparations across all strain × disc-content conditions (n = 44 per extract; based on Supplementary Material File S4). The n-hexane extract was excluded because it did not yield large inhibition zones. Overall distribution differed significantly from uniformity (χ^2^ = 58.8663, *p* < 0.001).

Extracts’ Preparation	Largest	2nd Largest	3d Largest	Top-3 (n, %)	Other	Total
Aqueous	-	-	3	3 (6.8)	41	44
Ethanol 40%	5	10	8	23 (52.3)	21	44
Ethanol 60%	21	14	8	43 (97.7)	1	44
Enzymatic	7	12	5	24 (54.5)	30	44
Ethyl acetate	5	6	9	20 (45.5)	24	44
n-Butanol	6	2	11	19 (43.2)	25	44
Total	44	44	44	100	132	264

**Table 7:** Minimum inhibitory (MIC, mg/L) and minimum bactericidal concentrations (MBC, mg/L) of *C. autumnale* flower-derived extracts against the tested pathogenic isolates and reference strains.

ID Pathogen/ Reference Strain	Minimum Inhibitory Concentration (mg/L)							
	n-H	EtOAc	n-BuOH	A	E40	E60	ENZ	ANOVA *p*
*S. aureus* 78	12.5 ± 0 ^e^	1.56 ± 0 ^d^	0.78 ± 0 ^c^	3.125 ± 0 ^f^	0.39 ± 0 ^b^	0.0975 ± 0 ^a^	0.0975 ± 0 ^a^	3.97 × 10^−207^
*S. aureus* ATCC 25923	12.5 ± 0 ^f^	1.56 ± 0 ^d^	0.195 ± 0 ^b^	6.25 ± 0 ^e^	3125 ± 0 ^g^	0.78 ± 0 ^c^	0.0975 ± 0 ^a^	10^−210^
*K. pneumoniae* 18	6.25 ± 0 ^c^	3.125 ± 0 ^d^	1.56 ± 0 ^b^	6.25 ± 0 ^c^	6.25 ± 0 ^c^	0.78 ± 0 ^a^	0.78 ± 0 ^a^	9.84 × 10^−217^
*K. pneumoniae* 94	6.25 ± 0 ^d^	1.56 ± 0 ^c^	0.39 ± 0 ^a^	6.25 ± 0 ^d^	1.56 ± 0 ^c^	0.39 ± 0 ^a^	0.78 ± 0 ^b^	6.13 × 10^−209^
*K. pneumoniae* 109	3.125 ± 0 ^d^	0.39 ± 0 ^c^	0.195 ± 0 ^b^	3.125 ± 0 ^d^	0.39 ± 0 ^c^	0.0975 ± 0 ^a^	0.39 ± 0 ^c^	9.49 × 10^−209^
*K. pneumoniae* 181	6.25 ± 0 ^b^	6.25 ± 0 ^b^	1.56 ± 0 ^a^	12.5 ± 0 ^c^	6.25 ± 0 ^b^	1.56 ± 0 ^a^	3.125 ± 0 ^d^	6.31 × 10^−215^
*K. pneumoniae* 328	6.25 ± 0 ^e^	1.56 ± 0 ^d^	0.195 ± 0 ^b^	3.125 ± 0 ^f^	1.56 ± 0 ^d^	0.0975 ± 0 ^a^	0.39 ± 0 ^c^	2.4 × 10^−207^
*K. pneumoniae* ATCC 700603	1.56 ± 0 ^e^	0.39 ± 0 ^c^	0.0975 ± 0 ^a^	0.78 ± 0 ^d^	0.39 ± 0 ^c^	0.0975 ± 0 ^a^	0.195 ± 0 ^b^	9.55 × 10^−211^
*P. aerigunosa* 40	6.25 ± 0 ^d^	0.195 ± 0 ^b^	0.195 ± 0 ^b^	6.25 ± 0 ^d^	1.56 ± 0 ^c^	0.0975 ± 0 ^a^	1.56 ± 0 ^c^	2.8 × 10^−209^
*P. aerigunosa* 309	ND	0.78 ± 0 ^b^	0.195 ± 0 ^a^	ND	6.25 ± 0 ^c^	0.195 ± 0 ^a^	3.125 ± 0 ^d^	9.81 × 10^−152^
*P. aeruginosa* ATCC 27853	3.125 ± 0 ^e^	1.56 ± 0 ^d^	0.195 ± 0 ^b^	3.125 ± 0 ^e^	3.125 ± 0 ^e^	0.0975 ± 0 ^a^	0.39 ± 0 ^c^	4.82 × 10^−215^
	**Minimum Bactericidal Concentration** **(mg/L)**							
*S. aureus* 78	12.5 ± 0 ^f^	6.25 ± 0 ^e^	1.56 ± 0 ^d^	3125 ± 0 ^g^	0.78 ± 0 ^c^	0.195 ± 0 ^b^	0.0975 ± 0 ^a^	1.95 × 10^−207^
*S. aureus* ATCC 25923	12.5 ± 0 ^d^	6.25 ± 0 ^c^	0.195 ± 0 ^a^	6.25 ± 0 ^c^	3.125 ± 0 ^e^	0.78 ± 0 ^b^	0.195 ± 0 ^a^	1.01 × 10^−210^
*K. pneumoniae* 18	12.5 ± 0 ^d^	3.125 ± 0 ^e^	3.125 ± 0 ^e^	6.25 ± 0 ^c^	6.25 ± 0 ^c^	0.78 ± 0 ^a^	1.56 ± 0 ^b^	1.04 × 10^−219^
*K. pneumoniae* 94	6.25 ± 0 ^c^	3.125 ± 0 ^d^	0.78 ± 0 ^b^	6.25 ± 0 ^c^	3.125 ± 0 ^d^	0.39 ± 0 ^a^	3125 ± 0 ^d^	3.63 × 10^−210^
*K. pneumoniae* 109	6.25 ± 0 ^e^	0.78 ± 0 ^d^	0.195 ± 0 ^b^	3.125 ± 0 ^f^	0.39 ± 0 ^c^	0.0975 ± 0 ^a^	0.39 ± 0 ^c^	4.46 × 10^−207^
*K. pneumoniae* 181	6.25 ± 0 ^a^	6.25 ± 0 ^a^	6.25 ± 0 ^a^	12.5 ± 0 ^b^	6.25 ± 0 ^a^	3.125 ± 0 ^c^	6.25 ± 0 ^a^	9.43 × 10^−215^
*K. pneumoniae* 328	6.25 ± 0 ^d^	3.125 ± 0 ^e^	0.195 ± 0 ^b^	3125 ± 0 ^e^	1.56 ± 0 ^c^	0.0975 ± 0 ^a^	1.56 ± 0 ^c^	7.33 × 10^−209^
*K. pneumoniae* ATCC 700603	3.125 ± 0 ^e^	0.39 ± 0 ^c^	0.0975 ± 0 ^a^	0.78 ± 0 ^d^	0.39 ± 0 ^c^	0.0975 ± 0 ^a^	0.195 ± 0 ^b^	2.1 × 10^−214^
*P. aerigunosa* 40	3.125 ± 0 ^d^	0.195 ± 0 ^a^	0.195 ± 0 ^a^	6.25 ± 0 ^c^	3.125 ± 0 ^d^	0.195 ± 0 ^a^	1.56 ± 0 ^b^	1.83 × 10^−212^
*P. aerigunosa* 309	ND	3.125 ± 0 ^c^	0.195 ± 0 ^a^	ND	6.25 ± 0 ^b^	0.195 ± 0 ^a^	3125 ± 0 ^c^	3.01 × 10^−154^
*P. aeruginosa* ATCC 27853	6.25 ± 0 ^c^	3.125 ± 0 ^d^	0.195 ± 0 ^a^	6.25 ± 0 ^c^	3.125 ± 0 ^d^	0.195 ± 0 ^a^	0.39 ± 0 ^b^	5.19 × 10^−211^

Notes: Values are presented as mean ± SD of three independent replicates (n = 3). Within each bacterial strain (row), MIC and MBC were analyzed separately among extracts using one-way ANOVA followed by Tukey’s HSD multiple-comparisons test (*p* < 0.05). Different superscript lowercase letters indicate significant differences among extracts within the same row for the respective endpoint (MIC or MBC); values sharing at least one letter are not significantly different. “ND”: Not determined.

**Table 8:** Minimum inhibitory (MIC, mg/L) and minimum bactericidal concentrations (MBC, mg/L) of *R. canina* pseudo-fruit-derived extracts against the tested pathogenic isolates and reference strains.

ID Pathogen/ Reference Strain	Minimum Inhibitory Concentration (mg/L)							
	n-H	EtOAc	n-BuOH	A	E40	E60	ENZ	ANOVA *p*
*S. aureus* 78	6.25 ± 0 ^f^	0.78 ± 0 e	0.195 ± 0 ^c^	0.39 ± 0 ^d^	0.39 ± 0 ^b^	0.0975 ± 0 ^b^	0.04875 ± 0 ^a^	9.13 × 10^−215^
*S. aureus* ATCC 25923	0.78 ± 0 ^e^	0.39 ± 0 ^d^	0.195 ± 0 ^c^	0.195 ± 0 ^c^	0.195 ± 0 ^c^	0.04875 ± 0 ^a^	0.0975 ± 0 ^b^	9.26 × 10^−213^
*K. pneumoniae* 18	6.25 ± 0 ^b^	6.25 ± 0 ^b^	6.25 ± 0 ^b^	3.125 ± 0 ^c^	0.195 ± 0 ^a^	0.195 ± 0 ^a^	3.125 ± 0 ^c^	1.25 × 10^−208^
*K. pneumoniae* 94	3.125 ± 0 ^d^	1.56 ± 0 ^c^	1.56 ± 0 ^c^	3.125 ± 0 ^d^	0.0975 ± 0 ^a^	0.0975 ± 0 ^a^	0.195 ± 0 ^b^	6.13 × 10^−209^
*K. pneumoniae* 109	1.56 ± 0 ^d^	0.195 ± 0 ^b^	0.39 ± 0 ^c^	0.39 ± 0 ^c^	0.195 ± 0 ^b^	0.0975 ± 0 ^a^	0.39 ± 0 ^c^	5.25 × 10^−213^
*K. pneumoniae* 181	6.25 ± 0 ^f^	6.25 ± 0 ^a^	6.25 ± 0 ^a^	6.25 ± 0 ^a^	6.25 ± 0 ^a^	3.125 ± 0 ^b^	6.25 ± 0 ^a^	9.76 × 10^−215^
*K. pneumoniae* 328	6.25 ± 0 ^f^	0.78 ± 0 e	0.78 ± 0 e	1.56 ± 0 ^d^	1.56 ± 0 ^d^	0.195 ± 0 ^a^	0.39 ± 0 ^b^	1.92 × 10^−213^
*K. pneumoniae* ATCC 700603	0.39 ± 0 ^c^	0.78 ± 0 ^d^	0.78 ± 0 ^d^	0.195 ± 0 ^b^	0.195 ± 0 ^b^	0.0975 ± 0 ^a^	0.0975 ± 0 ^a^	1.82 × 10^−217^
*P. aerigunosa* 40	3.125 ± 0 ^f^	0.195 ± 0 ^b^	0.39 ± 0 ^c^	6.25 ± 0 ^e^	0.0975 ± 0 ^a^	0.195 ± 0 ^b^	0.78 ± 0 ^d^	1.03 × 10^−197^
*P. aerigunosa* 309	3.125 ± 0 ^d^	0.39 ± 0 ^b^	0.39 ± 0 ^b^	3.125 ± 0 ^d^	0.78 ± 0 ^c^	0.195 ± 0 ^a^	3.125 ± 0 ^d^	4.79 × 10^−209^
*P. aeruginosa* ATCC 27853	3.125 ± 0 ^d^	0.78 ± 0 ^e^	0.195 ± 0 ^a^	3.125 ± 0 ^d^	3.125 ± 0 ^d^	0.78 ± 0 ^b^	1.56 ± 0 ^c^	1.79 × 10^−214^
	Minimum Bactericidal Concentration (mg/L)							
*S. aureus* 78	6.25 ± 0 ^d^	1.56 ± 0 ^c^	0.39 ± 0 ^b^	0.39 ± 0 ^b^	0.39 ± 0 ^b^	0.0975 ± 0 ^a^	0.0975 ± 0 ^a^	9.91 × 10^−215^
*S. aureus* ATCC 25923	0.78 ± 0 ^e^	0.39 ± 0 ^d^	0.195 ± 0 ^c^	0.39 ± 0 ^d^	0.195 ± 0 ^c^	0.04875 ± 0 ^a^	0.0975 ± 0 ^b^	5.23 × 10^−212^
*K. pneumoniae* 18	6.25 ± 0 ^b^	6.25 ± 0 ^b^	6.25 ± 0 ^b^	6.25 ± 0 ^b^	0.195 ± 0 ^a^	0.195 ± 0 ^a^	3125 ± 0 ^c^	1.7 × 10^−215^
*K. pneumoniae* 94	6.25 ± 0 ^e^	3.125 ± 0 ^f^	1.56 ± 0 ^d^	3.125 ± 0 ^f^	0.0975 ± 0 ^a^	0.195 ± 0 ^b^	0.39 ± 0 ^c^	4.7 × 10^−209^
*K. pneumoniae* 109	1.56 ± 0 ^d^	0.78 ± 0 ^c^	0.39 ± 0 ^b^	0.39 ± 0 ^b^	0.195 ± 0 ^a^	0.195 ± 0 ^a^	0.78 ± 0 ^c^	6.94 × 10^−213^
*K. pneumoniae* 181	6.25 ± 0 ^a^	6.25 ± 0 ^a^	6.25 ± 0 ^a^	6.25 ± 0 ^a^	6.25 ± 0 ^a^	6.25 ± 0 ^a^	6.25 ± 0 ^a^	0.563
*K. pneumoniae* 328	6.25 ± 0 ^d^	0.78 ± 0 ^c^	3.125 ± 0 ^e^	3.125 ± 0 ^e^	3.125 ± 0 ^e^	0.195 ± 0 ^a^	0.39 ± 0 ^b^	1.28 × 10^−209^
*K. pneumoniae* ATCC 700603	0.78 ± 0 ^d^	0.78 ± 0 ^d^	1.56 ± 0 ^e^	0.39 ± 0 ^c^	0.195 ± 0 ^b^	0.0975 ± 0 ^a^	0.0975 ± 0 ^a^	7.68 × 10^−215^
*P. aerigunosa* 40	3.125 ± 0 ^e^	0.195 ± 0 ^b^	0.78 ± 0 ^c^	6.25 ± 0 ^d^	0.0975 ± 0 ^a^	0.195 ± 0 ^b^	0.78 ± 0 ^c^	7.68 × 10^−215^
*P. aerigunosa* 309	6.25 ± 0 ^d^	0.39 ± 0 ^b^	0.0.78 ± 0 ^c^	3.125 ± 0 ^e^	0.78 ± 0 ^c^	0.195 ± 0 ^a^	0.78 ± 0 ^c^	2.65 × 10^−194^
*P. aeruginosa* ATCC 27853	1.56 ± 0 ^b^	1.56 ± 0 ^b^	0.195 ± 0 ^a^	6.25 ± 0 ^c^	6.25 ± 0 ^c^	1.56 ± 0 ^b^	1.56 ± 0 ^b^	1.36 × 10^−208^

Notes: Values are presented as mean ± SD of three independent replicates (n = 3). Within each bacterial strain (row), MIC and MBC were analyzed separately among extracts using one-way ANOVA followed by Tukey’s HSD multiple-comparisons test (*p* < 0.05). Different superscript lowercase letters indicate significant differences among extracts within the same row for the respective endpoint (MIC or MBC); values sharing at least one letter are not significantly different. “ND”: Not determined.

**Table 9:** Comparative distribution of the principal tentatively annotated phytochemicals in the selected *Colchicum autumnale* L. flower (CAE/E60, CAE/ENZ) and *Rosa canina* pseudo-fruit extracts (WFE/E60, WFE/ENZ) by UHPLC-DAD-MS/MS.

a/a	Compound Name	Nature of Compound	Molecular Formula	Present		Compound Name	Nature of Compound	Molecular Formula	Present	
				CAE/ E60	CAE/ ENZ				WFE/ E60	WFE/ ENZ
1	Colchicine	Tropolone alkaloid	C_22_H_25_NO_6_	+	+	Kaempferol	Flavonol	C_15_H_10_O_6_	+	+
2	Demethylcolchicine/colchiceine-type isomer 1	Colchicine-type tropolone alkaloid	C_21_H_23_NO_6_	+	-	Luteolin	Flavone	C_15_H_10_O_6_	+	+
3	Colchiceine	Tropolone alkaloid derivative	C_21_H_23_NO_6_	+	+	Quercetin	Flavonol	C_15_H_10_O_7_	+	+
4	Colchicoside	Tropolone alkaloid glycoside	C_27_H_33_NO_11_	-	+	Rutin (quercetin-3-O-rutinoside)	Flavonol glycoside	C_27_H_30_O_16_	+	+
5	Demecolcine	Tropolone alkaloid derivative	C_21_H_25_NO_5_	+	+	Catechin/epicatechin-type flavan-3-ol (1)	Flavonoid; flavan-3-ol/flavanol	C_15_H_14_O_6_	+	+
6	Colchiciline	Tropolone alkaloid derivative	C_22_H_25_NO_7_	+	-	Catechin/epicatechin-type flavan-3-ol (2)	Flavonoid; flavan-3-ol/flavanol	C_15_H_14_O_6_	+	+
7	Deacetamido-5,6-dihydrocolchicine	Colchicine-type tropolone derivative	C_20_H_20_O_5_	+	-	Quercetin glycoside-type compound (possible co-eluting quercetin glycosides	Flavonoid/flavonol glycoside	-	+	+
8	Demethylcolchicine/colchiceine-type isomer 2	Colchicine-type tropolone alkaloid	C_21_H_23_NO_6_	+	-	Kaempferol 3-O-rutinoside	Flavonol glycoside	C_27_H_30_O_15_	+	+
9	Syringic acid	Phenolic acid	C_9_H_10_O_5_	+	+	Kaempferol glycoside-type compound (possible co-eluting kaempferol glycosides)	Flavonoid/flavonol glycoside	-	+	+
10	Vanillic acid	Phenolic acid	C_8_H_8_O_4_	-	+	Naringenin	Flavanone	C_15_H_12_O_5_	+	-
11	Ferulic acid	Hydroxycinnamic acid	C_10_H_10_O_4_	+	+	Naringenin-7-O-glucoside	Flavanone glycoside	C_21_H_22_O_10_	-	+
12	Caffeic acid	Hydroxycinnamic acid	C_9_H_8_O_4_	-	+	Hesperidin/flavanone glycoside	Flavanone glycoside	C_28_H_34_O_15_	-	+
13	Rosmarinic acid	Phenolic ester	C_18_H_16_O_8_	-	+	Ellagic-acid derivative	Tannin-related phenolic/ellagic acid derivative	-	-	+
14	Apigenin	Flavone	C_15_H_10_O_5_	+	+	Quinic acid	Organic acid	C_7_H_12_O_6_	+	+
15	Luteolin	Flavone	C_15_H_10_O_6_	-	+	Citric acid	Organic acid	C_6_H_8_O_7_	+	+
16	Kaempferol	Flavonol	C_15_H_10_O_6_	+	-	Caffeic acid	Hydroxycinnamic acid	C_9_H_8_O_4_	+	+
17	Coumaric acid isomer	Hydroxycinnamic acid derivative	C_9_H_8_O_3_	+	+	Ferulic acid	Hydroxycinnamic acid	C_10_H_10_O_4_	+	+
18	Catechin/epicatechin-type flavan-3-ol	Flavonoid; flavan-3-ol/flavanol	C_15_H_14_O_6_	+	+	Coumaric acid isomer	Phenolic acid; hydroxycinnamic acid derivative	C_9_H_8_O_3_	+	+
19	Matairesinol	Lignan	C_20_H_22_O_6_	-	+	Vanillic acid	Phenolic acid	C_8_H_8_O_4_	+	+
20	Not identified (Unknown compound 1)	-	-	+	-	Syringic acid	Phenolic acid	C_9_H_10_O_5_	-	+
21	Not identified (Unknown compound 2)	-	-	-	+	Protocatechuic acid	Phenolic acid	C_7_H_6_O_4_	-	+
22	Not identified (Unknown compound 3)	-	-	+	-	Salicylic acid	Phenolic acid	C_7_H_6_O_3_	+	+
23						Procyanidin B-type dimer/procyanidin-type oligomer	Flavonoid; condensed tannin/proanthocyanidin	-	-	+
24						Rosmarinic acid	Phenolic ester	C_18_H_16_O_8_	+	-
25						Resveratrol	Stilbene polyphenol	C_14_H_12_O_3_	+	-
26						Not identified (Unknown compound 1)	-	-	+	-
27						Not identified (Unknown compound 2)	-	-	-	+

**Table 10:** Qualitative tests for phenotypic detection of major classes of bioactive molecules in CA (*C. autumnale* flower) and WF (*R. canina* pseudo-fruit) extracts.

Target Class Classes of Secondary Metabolites	Test/Reagent (Principle)	Visual Endpoint Recorded	Key References
Alkaloids	Dragendorff’s and/or Mayer’s test (acid extract + BiI_4_^−^/K_2_HgI_4_)	Orange–brown (Dragendorff) or cream (Mayer) precipitate	[86,87,88]
Anthraquinones	Bornträger reaction (extract + H_2_SO_4_, then NH_4_OH)	Pink to red colour in alkaline (ammonia) phase	[89,90]
Terpenoids	Salkowski test (chloroform + conc. H_2_SO_4_)	Reddish-brown or violet ring at the interface	[91,92,93]
Steroids	Liebermann–Burchard reaction (acetic anhydride + H_2_SO_4_)	Blue–green to emerald colour	[93]
Saponins	Froth test (vigorous shaking in water)	Stable persistent foam (≥10–15 min)	[83,84,90]
Tannins	Ferric chloride test (3–5% FeCl_3_ in ethanol) (Braymer’s test)	Blue–black or greenish-black colour	[94,95]
Cardiac glycosides	Keller–Kiliani test (glacial acetic acid + FeCl_3_, underlay with H_2_SO_4_)	Brown ring at interface and bluish-green upper layer	[96,97]
“Total phenolics” (qualitative)	Folin–Ciocalteu reaction (FC reagent + Na_2_CO_3_)	Blue to blue–green colour vs. solvent blank	[94]
Flavonoids	Alkaline reagent test (NaOH, then dilute HCl)	Intense yellow colour that disappears on acidification	[95,98]
Carotenoids	Hexane/acetone partition (or petroleum ether layer)	Yellow–orange colour in non-polar layer	[89,99]
Ascorbic acid (vitamin C)	DCPIP (2,6-dichlorophenol-indophenol) reduction test	Rapid decolourisation of blue DCPIP solution	[94]

## Data Availability

The original contributions presented in this study are included in the article/Supplementary Material. Further inquiries can be directed to the corresponding author.

## Supplementary Materials

The following supporting information can be downloaded at: https://www.mdpi.com/article/10.3390/antibiotics15050508/s1, Supplementary Material File S1: Percentage neutralization of the DPPH radical by solvent-partitioned fractions from *C. autumnale* flowers (CA) and *R. canina* pseudo-fruits (WF) across a concentration gradient (mean ± SD, n = 3); Supplementary Material File S2: Ferric-reducing antioxidant power (FRAP) of solvent-partitioned fractions from *C. autumnale* flowers (CA) and *R. canina* pseudo-fruits (WF), expressed as absorbance values across the tested concentration range (mean ± SD, n = 3); Supplementary Material File S3: Inhibition zone diameters produced *by C. autumnale* flower-derived extracts at different concentrations (10, 20, 50, and 100%) against pathogenic isolates and reference strains; Supplementary Material File S4: Inhibition zone diameters produced by *R. canina* pseudo-fruit (WF)–derived extracts at different concentrations (10, 20, 50, and 100%) against pathogenic isolates and reference strains; Supplementary Material File S5: Tentatively annotated compounds detected by UHPLC-DAD-MS/MS in the 60% hydroethanolic extract (CAE/E60) and enzyme-assisted extract (CAE/ENZ) of *Colchicum autumnale* L. flowers; Supplementary Material File S6: Tentatively annotated compounds detected by UHPLC-DAD-MS/MS in the 60% hydroethanolic extract (WFE/E60) and enzyme-assisted extract (WFE/ENZ) of *Rosa canina* pseudo-fruits.
