# Supplementary material for: Evaluation of Plant-Guided Strategies Against Clinical Multidrug-Resistant Pathogens: Preliminary Phytochemical Screening, Antioxidant Capacity, and Antibacterial/Antibiofilm Activity of Rosa canina and Colchicum autumnale Extracts
Source: Antibiotics (Basel). 2026 May 18;15(5):508. doi: 10.3390/antibiotics15050508 (PMC13203422; doi:10.3390/antibiotics15050508)
Supplement: Supplementary file 1 [file antibiotics-15-00508-s001.zip › S1.pdf]

Concentration (µg/mL)	*Colchicum autumnale* flower fractions (CA)								*Rosa canina* pseudo-fruit fractions (WF)							
	n-H	EtOAc	n-BuOH	A	E40	E60	ENZ	Kruskal–Wallis p	n-H	EtOAc	n-BuOH	A	E40	E60	ENZ	Kruskal–Wallis p
500	46.1 ± 1.1^{d}	88.3 ± 1.4^{b}	91.2 ± 1.4^{a}	61.0 ± 1.1^{d}	77.4 ± 0.4^{c}	87.9 ± 0.2^{b}	82.9 ± 0.9^{c}	0.003649	37.9 ± 1.1d	88.3 ± 0.4^{c}	86.3 ± 0.4^{cd}	90.8 ± 0.9^{b}	95.5 ± 0.6^{a}	91.5 ± 0.6^{b}	95.6 ± 0.5^{a}	0.004071
300	19.6 ± 1.0^{d}	79.4 ± 0.3^{a}	83.5 ± 1.2^{a}	47.8 ± 0.5^{cd}	64.8 ± 1.5^{c}	75.1 ± 0.7^{b}	74.6 ± 0.7^{b}	0.003663	24.7 ± 1.3d	81.8 ± 0.8^{c}	79.3 ± 0.6^{cd}	84.5 ± 0.7^{b}	87.8 ± 0.4^{a}	86.4 ± 1.5^{b}	90.9 ± 0.2^{a}	0.003709
100	14.8 ± 0.6^{g}	67.7 ± 0.5^{c}	78.2 ± 0.7^{a}	45.9 ± 0.4^{f}	61.0 ± 0.5^{e}	66.2 ± 0.3^{d}	73.0 ± 0.5^{b}	0.003214	21.1 ± 0.5d	77.3 ± 0.5^{b}	66.3 ± 0.7^{cd}	69.6 ± 0.6^{c}	77.8 ± 0.6^{b}	78.6 ± 0.5^{a}	80.4 ± 1.0^{a}	0.003683
50	7.9 ± 0.2^{g}	41.4 ± 0.8^{d}	44.8 ± 0.8^{c}	19.3 ± 0.5^{f}	33.3 ± 0.6^{e}	48.2 ± 0.5^{b}	64.8 ± 2.5^{a}	9.1 ± 0.2d	9.1 ± 0.2^{d}	54.2 ± 1.4^{c}	51.9 ± 1.2^{cd}	58.7 ± 0.4^{b}	61.0 ± 0.3^{a}	58.8 ± 0.2^{b}	64.7 ± 0.4^{a}	0.003775
10	2.3 ± 0.4^{d}	12.4 ± 0.5^{b}	14.2 ± 0.4^{a}	6.6 ± 0.6^{cd}	11.3 ± 0.5^{c}	13.5 ± 0.5^{b}	13.6 ± 0.5^{a}	0.004081	4.0 ± 0.1^{g}	21.3 ± 0.4^{e}	19.2 ± 0.3^{f}	22.5 ± 0.4^{d}	26.1 ± 0.3^{b}	25.0 ± 0.4^{c}	28.8 ± 0.7^{a}	0.003197
5	1.7 ± 0.2^{b}	9.0 ± 1.0^{a}	8.7 ± 0.3^{a}	4.4 ± 0.3^{b}	6.7 ± 0.4^{b}	7.9 ± 0.2^{a}	8.2 ± 0.3^{a}	0.006583	2.6 ± 0.3^{d}	12.8 ± 0.6^{c}	12.3 ± 0.4^{cd}	15.1 ± 0.5^{b}	17.6 ± 0.6^{a}	16.0 ± 0.6^{b}	18.9 ± 0.2^{a}	0.003753
2.5	1.1 ± 0.1^{b}	4.9 ± 0.5^{a}	6.0 ± 0.7^{a}	2.5 ± 0.4^{b}	3.7 ± 0.2^{b}	4.8 ± 0.6^{a}	5.7 ± 0.4^{a}	0.005767	1.9 ± 0.4^{c}	7.9 ± 0.2^{b}	6.7 ± 0.2^{c}	9.2 ± 0.3^{b}	9.8 ± 0.4^{a}	10.2 ± 0.9^{a}	11.2 ± 1.0^{a}	0.004641
1	0.5 ± 0.1^{b}	3.0 ± 0.1^{a}	2.7 ± 0.2^{a}	1.9 ± 0.3^{b}	2.3 ± 0.3^{b}	2.8 ± 0.1^{a}	2.6 ± 0.2^{a}	0.008942	1.4 ± 0.1^{c}	3.8 ± 0.3^{b}	3.5 ± 0.2^{c}	4.3 ± 0.3^{b}	4.7 ± 0.2^{a}	4.7 ± 0.2^{a}	5.8 ± 0.3^{a}	0.005099

Table S1: Percentage neutralization of the DPPH radical by solvent-partitioned fractions from *C. autumnale* flowers (CA) and *R. canina* pseudo-fruits (WF) across a concentration gradient (mean ± SD, n = 3). Different superscript letters in columns denote statistical differences among the various extracts in each plant (Kruskal–Wallis, $p < 0.05$ with Tukey's HSD)
