# Supplementary material for: Evaluation of Plant-Guided Strategies Against Clinical Multidrug-Resistant Pathogens: Preliminary Phytochemical Screening, Antioxidant Capacity, and Antibacterial/Antibiofilm Activity of Rosa canina and Colchicum autumnale Extracts
Source: Antibiotics (Basel). 2026 May 18;15(5):508. doi: 10.3390/antibiotics15050508 (PMC13203422; doi:10.3390/antibiotics15050508)
Supplement: Supplementary file 1 [file antibiotics-15-00508-s001.zip › S6.pdf]

No. Peaks	Compound name	t_R (min)	Precursor ion (m/z)	Main MS/MS fragments	Peak purity (%)	Presence in Sample	
						WFE/E60	WFE/ENZ
1	Quinic acid	0.75	191 [M-H]-	173, 127, 111, 85	91	+	+
2	Citric acid	0.92	191 [M-H]-	173, 111	92	+	+
3	Caffeic acid	1.02	179 [M-H]-	161, 143, 131, 119, 89	90	+	+
4	Vanillic acid	1.14	167 [M-H]- / 169 [M+H]+	152, 123, 108	92	+	+
5	Syringic acid	1.20	197 [M-H]- / 199 [M+H]+	182, 167, 153, 123	97	-	+
6	Protocatechuic acid	1.31	153 [M-H]- / 155 [M+H]+	109, 91	92	-	+
7	Salicylic acid	1.41	137 [M-H]- / 139 [M+H]+	93, 65	92	+	+
8	Ferulic acid	1.53	193 [M-H]-	178, 149, 134	90	+	+
9	Coumaric acid isomer	2.08	163 [M-H]-	119	91	+	+
10	Catechin/epicatechin-type flavan-3-ol (1)	3.89	289 [M-H]- / 291 [M+H]+	245, 205, 179, 123	90	+	+
11	Catechin/epicatechin-type flavan-3-ol (2)	4.05	289 [M-H]- / 291 [M+H]+	245, 205, 179, 123	90	+	+
12	Rosmarinic acid	4.79	359 [M-H]-	197, 179, 161, 135	95	+	-
13	Procyanidin B-type dimer / procyanidin-type oligomer	5.02	577 [M-H]- / 579 [M+H]+	451, 425, 407, 289	90	-	+
14	Quercetin glycoside-type compound (possible co-eluting quercetin glycosides)	7.12	463 & 609 [M-H]-	301, 300, 299, 271	91	+	+
15	Kaempferol glycoside-type compound (possible co-eluting kaempferol glycosides)	7.19	447 & 593 [M-H]-	285, 255	90	+	+
16	Kaempferol 3-O-rutinoside	7.21	593 [M-H]- / 595 [M+H]+	285, 255	95	+	+
17	Rutin (quercetin-3-O-rutinoside)	7.25	609 [M-H]-	301, 299, 271	95	+	+
18	Not identified (Unknown 1)	7.38	389 [M-H]-	277, 148, 127	91	+	-
19	Naringenin-7-O-glucoside	7.44	433 [M-H]- / 435 [M+H]+	271, 151, 119	90	-	+
20	Hesperidin / flavanone glycoside-type compound	7.59	609 [M-H]- / 611 [M+H]+	301, 286, 255	90	-	+
21	Luteolin	8.07	285 [M-H]-	151, 133	90	+	+
22	Kaempferol	8.86	285 [M-H]- / 287 [M+H]+	255, 227, 151	95	+	+
23	Quercetin	9.08	301 [M-H]- / 303 [M+H]+	179, 151, 121	94	+	+
24	Not identified (Unknown 2)	9.70	604 [M−H]−	489, 301	92	-	+
25	Ellagic acid / ellagic-acid derivative	9.77	301 [M-H]-	257, 229, 185	90	-	+
26	Naringenin	11.40	271 [M-H]-	227, 177, 151, 107, 93	94	+	-
27	Resveratrol	11.88	227 [M-H]- / 229 [M+H]+	185, 159, 143	90	+	-

Table S6: Tentatively annotated compounds detected by UHPLC-DAD-MS/MS in the 60% hydroethanolic extract (WFE/E60) and enzyme-assisted extract (WFE/ENZ) of *Rosa canina* pseudo-fruits

Abbreviations: tR, retention time in min; MS/MS, tandem mass spectrometry; WFE/E60, 60% hydroethanolic extract; WFE/ENZ, enzyme-assisted extract; (+) and (-) signs indicate detection or non-detection under the applied analytical conditions; Peak purity (%): the estimated chromatographic spectral homogeneity of the detected peak, expressed as a percentage, as calculated by the instrument/software from DAD and/or MS signal evaluation
