# Supplementary material for: Evaluation of Plant-Guided Strategies Against Clinical Multidrug-Resistant Pathogens: Preliminary Phytochemical Screening, Antioxidant Capacity, and Antibacterial/Antibiofilm Activity of Rosa canina and Colchicum autumnale Extracts
Source: Antibiotics (Basel). 2026 May 18;15(5):508. doi: 10.3390/antibiotics15050508 (PMC13203422; doi:10.3390/antibiotics15050508)
Supplement: Supplementary file 1 [file antibiotics-15-00508-s001.zip › S5.pdf]

No. Peaks	Compound name	t_R (min)	Precursor ion (m/z)	Main MS/MS fragments pattern	Peak purity (%)	Presence in Sample	
						CAE/E60	CAE/ENZ
1	Caffeic acid	0.98	179 [M-H]-	161, 143, 131, 119, 89	90%	-	+
2	Vanillic acid	1.15	167 [M-H]- / 169 [M+H]+	152, 123, 108	92	-	+
3	Syringic acid	1.22	197 [M-H]- / 199 [M+H]+	182, 167, 153, 123	97	+	+
4	Ferulic acid	1.51	193 [M-H]-	178, 149, 134	90	+	+
5	Coumaric acid isomer	2.04	163 [M-H]-	119	91	+	+
6	Not identified (Unknown compound 1)	2.51	301 [M-H]-	191, 179	92	+	-
7	Catechin/epicatechin-type flavan-3-ol	3.88	289 [M-H]- / 291 [M+H]+	245, 205, 179, 123	90	+	+
8	Rosmarinic acid	4.81	359 [M-H]-	197, 179, 161, 135	90	-	+
9	Matairesinol	5.10	357 [M-H]- / 359 [M+H]+	342, 221, 151, 137	90	-	+
10	Colchicoside	5.80	548.212 [M+H]+	405, 386, 368, 344, 295, 267	95	-	+
11	Deacetamido-5,6-dihydrocolchicine	6.10	341.138 [M+H]+	264.9, 232.9, 214.9, 167.0	97	+	-
12	Demethylcolchicine/colchiceine-type isomer 1	6.32	386.159 [M+H]+	368.147, 344.149, 295.096, 250.939, 207.080	95	+	-
13	Demethylcolchicine/colchiceine-type isomer 2	6.57	386.159 [M+H]+	344, 295	96	+	-
14	Demecolcine	6.83	372.180 [M+H]+	340, 325	97	+	+
15	Colchiciline	7.19	416 [M+H]+	358, 136, 108	95	+	+
16	Not identified (Unknown compound 2)	7.32	414 [M-H]- / 416 [M+H]+	321, 358, 167	90	-	+
17	Colchicine	7.77	400.175 [M+H]+	382, 368, 358, 287, 114	96	+	+
18	Luteolin	8.02	285 [M-H]-	151, 133	90	-	+
19	Kaempferol	8.89	285 [M-H]- / 287 [M+H]+	255, 227, 151	90	+	-
20	Not identified (Unknown compound 3)	9.42	571 [M-H]-	449	90	+	-
21	Apigenin	9.80	269 [M-H]- / 271 [M+H]+	151, 149, 117	94	+	+
22	Colchiceine	17.70	386.160 [M+H]+	344, 296	96	+	+

Table S5: Tentatively annotated compounds detected by UHPLC-DAD-MS/MS in the 60% hydroethanolic extract (CAE/E60) and enzyme-assisted extract (CAE/ENZ) of *Colchicum autumnale L.* flowers

Abbreviations: t_R, retention time in min; MS/MS, tandem mass spectrometry; CAE/E60, 60% hydroethanolic extract; CAE/ENZ, enzyme-assisted extract; (+) and (-) signs indicate detection or non-detection under the applied analytical conditions; Peak purity (%): the estimated chromatographic spectral homogeneity of the detected peak, expressed as a percentage, as calculated by the instrument/software from DAD and/or MS signal evaluation
