# Supplementary material for: Evaluation of Plant-Guided Strategies Against Clinical Multidrug-Resistant Pathogens: Preliminary Phytochemical Screening, Antioxidant Capacity, and Antibacterial/Antibiofilm Activity of Rosa canina and Colchicum autumnale Extracts
Source: Antibiotics (Basel). 2026 May 18;15(5):508. doi: 10.3390/antibiotics15050508 (PMC13203422; doi:10.3390/antibiotics15050508)
Supplement: Supplementary file 1 [file antibiotics-15-00508-s001.zip › S4.pdf]

ID Pathogen/ Reference strain	Disk content	*Rosa canina* pseudo-fruit fractions (WF)						
		A	E40	E60	ENZ	n-H	EtOAc	n-BuOH
S. aureus 78	10%	7.44 ± 0.23	10.27 ± 0.16	14.33 ± 0.42	16.71 ± 0.42	6.01 ± 0.01	6.07 ± 0.04	10.58 ± 0.17
	20%	8.70 ± 0.35	15.25 ± 1.10	19.24 ± 0.24	25.97 ± 0.45	6.09 ± 0.02	8.79 ± 0.11	26.25 ± 0.57
	50%	23.90 ± 0.19	20.34 ± 0.45	31.5 ± 1.23	43.53 ± 0.55	9.68 ± 0.27	17.58 ± 0.25	37.77 ± 0.22
	100%	26.43 ± 0.69	29.29 ± 0.39	52.01 ± 0.13	60.47 ± 1.41	10.35 ± 0.47	19.03 ± 0.37	44.48 ± 1.55
K. pneumoniae 18	10%	6.13 ± 0.01	9.77 ± 1.35	11.51 ± 0.50	10.46 ± 0.25	6.38 ± 0.43	7.45 ± 0.52	8.43 ± 3.1
	20%	8.45 ± 0.29	12.20 ± 0.35	14.08 ± 0.62	22.45 ± 0.15	7.34 ± 0.47	10.48± 0.37	7.75 ± 0.15
	50%	17.74 ± 0.46	24.94 ± 0.19	28.55 ± 0.58	25.23 ± 0.18	7.99 ± 0.19	12.67 ± 0.28	10.23 ± 0.13
	100%	19.70 ± 0.22	49.49 ± 0.62	44.50 ± 1.29	29.23 ± 0.25	7.94 ± 0.05	12.70 ± 0.29	12.62 ± 1.15
K. pneumoniae 94	10%	11.25 ± 1.05	11.71 ± 0.43	12.79 ± 0.65	9.54 ± 0.27	8.40 ± 0.33	13.36 ± 0.32	10.49 ± 0.39
	20%	15.96 ± 0.89	18.54 ± 0.44	17.98 ± 0.18	19.56 ± 0.32	8.57 ± 0.27	15.51 ± 1.38	11.65 ± 0.44
	50%	19.54 ± 0.42	26.11 ± 0.98	24.65 ± 0.72	20.75 ± 0.19	9.70 ± 0.23	24.42 ± 1.29	17.29 ± 3.72
	100%	19.16 ± 0.05	51.30 ± 1.43	49.35 ± 0.34	36.68 ± 1.97	17.37 ± 0.18	41.33 ± 1.35	26.15 ± 1.13
K. pneumoniae 109	10%	9.99 ± 0.22	12.35 ± 0.12	13.54 ± 0.22	10.65 ± 0.43	9.49 ± 0.53	10.74 ± 0.58	11.63 ± 0.78
	20%	13.39 ± 0.52	12.89 ± 0.09	35.38 ± 0.55	19.32 ± 1.36	12.54 ± 0.92	12.49 ± 0.49	14.13 ± 0.59
	50%	18.44 ± 0.68	35.62 ± 0.43	40.43 ± 0.36	21.12 ± 0.84	17.69 ± 0.36	15.57 ± 0.38	21.73 ± 0.16
	100%	26.40 ± 1.66	39.30 ± 0.44	42.91 ± 0.07	22.99 ± 0.55	19.71± 0.24	36.59 ± 1.97	29.89 ± 0.92
K.pneumoniae 181	10%	6.10 ± 0.10	7.16 ± 0.06	7.46 ± 0.34	6.30 ± 0.22	6.61 ± 0.46	6.26 ± 0.26	13.31 ± 0.45
	20%	7.30 ± 0.35	7.74 ± 0.28	8.91 ± 0.71	6.51 ± 0.30	6.41 ± 0.28	6.38 ± 0.32	6.55 ± 0.39
	50%	9.03 ± 0.93	10.82 ± 0.29	19.85 ± 1.01	7.88 ± 0.08	6.90 ± 0.03	10.69 ± 0.44	6.78 ± 0.52
	100%	10.39 ± 0.33	10.86 ± 0.12	19.97 ± 0.23	10.89 ± 0.04	9.55 ± 0.27	11.72 ± 0.30	7.08 ± 0.09
K.pneumoniae 328	10%	9.37 ± 0.45	11.16 ± 0.34	12.20 ± 0.34	10.53 ± 0.25	9.37 ± 0.38	14.02 ± 0.80	11.73 ± 0.62
	20%	18.14 ± 0.59	12.76 ± 0.36	14.25 ± 0.63	20.58 ± 0.37	10.14 ± 0.06	23.77 ± 0.17	14.22 ± 0.57
	50%	18.90 ± 0.04	14.84 ± 0.36	32.56 ± 0.38	22.35 ± 0.24	11.91 ± 0.04	26.56 ± 0.54	17.82 ± 1.25
	100%	20.35 ± 0.22	18.64 ± 0.37	36.88 ± 0.52	22.74 ± 0.08	11.92 ± 0.06	27.19 ± 0.82	24.51 ± 1.73
P. aeruginosa 40	10%	6.38 ± 0.23	13.63 ± 0.32	12.37 ± 1.71	8.94 ± 0.07	10.82 ± 0.17	12.70 ± 0.41	11.41 ± 0.36
	20%	6.68 ± 0.15	14.68 ± 0.13	20.13 ± 0.39	17.39 ± 4.54	10.87 ± 0.08	18.18 ± 0.48	21.99 ± 0.79
	50%	8.10 ± 0.95	28.53 ± 0.82	30.36 ± 0.27	22.09 ± 0.70	15.70 ± 1.46	28.01± 1.30	23.17 ± 0.08
	100%	9.62 ± 0.59	39.66 ± 0.12	32.83 ± 0.43	22.85 ± 0.12	18.67 ± 0.91	38.86 ± 0.63	24.85 ± 0.10
P. aeruginosa 309	10%	8.72 ± 0.57	10.63 ± 0.59	11.73 ± 0.71	10.21 ± 0.04	8.65 ± 0.35	10.28 ± 0.36	9.19 ± 0.08
	20%	10.60 ± 0.57	15.65 ± 0.46	18.68 ± 0.72	19.84 ± 0.71	10.63 ± 0.39	21.04 ± 0.44	12.69 ± 0.17
	50%	12.03 ± 0.78	25.93 ± 0.06	27.67 ± 0.39	20.94 ± 0.74	12.37 ± 0.19	27.87 ± 0.43	22.56 ± 0.29
	100%	16.15 ± 0.53	28.29 ± 0.15	34.04 ± 0.41	27.85 ± 0.43	16.61 ± 0.35	33.07 ± 0.78	29.23 ± 0.66
P. aeruginosa ATCC 27853	10%	8.73 ± 0.57	10.62 ± 0.58	11.73 ± 0.71	10.21 ± 0.04	9.03 ± 0.73	10.45 ± 0.37	12.45 ± 0.43
	20%	10.6 ± 0.93	15.65 ± 0.46	18.68 ± 0.72	19.84 ± 0.71	13.19 ± 0.34	15.01 ± 0.52	19.02 ± 0.49
	50%	12.03 ± 0.78	25.93 ± 0.06	27.67 ± 0.39	20.94 ± 0.74	19.23 ± 0.46	21.48 ± 0.63	30.50 ± 0.56
	100%	16.15 ± 0.53	26.29 ± 0.15	34.04 ± 0.41	27.85 ± 0.43	17.04 ± 4.43	18.69 ± 0.88	40.66 ± 0.79
S. aureus ATCC 29213	10%	13.58 ± 0.10	11.84 ± 0.16	17.92 ± 0.99	16.61 ± 1.14	7.84 ± 0.16	10.50 ± 0.33	10.34 ± 0.13
	20%	14.49 ± 0.23	19.00 ± 0.33	44.24 ± 0.57	26.23 ± 0.26	9.53 ± 0.30	18.89 ± 0.35	23.13 ± 0.42
	50%	25.89 ± 1.49	29.79 ± 0.11	61.05 ± 0.63	48.47 ± 0.82	16.91 ± 0.94	22.29 ± 0.65	34.68 ± 1.43
	100%	23.67 ± 0.22	30.79 ± 0.03	70.46 ± 0.63	60.75 ± 0.12	20.54 ± 0.11	29.57 ± 0.19	35.61 ± 0.17
K. quasipneumoniae ATCC 700603	10%	8.74 ± 0.63	12.21 ± 0.56	12.61 ± 0.48	11.71 ± 0.15	9.03 ± 0.70	10.23 ± 1.17	8.88 ± 0.46
	20%	10.46 ± 0.35	22.32 ± 0.65	20.28 ± 0.69	23.04 ± 0.12	12.25 ± 0.56	18.79 ± 1.14	12.88 ± 0.52
	50%	18.90 ± 0.74	33.19 ± 0.65	41.35 ± 0.71	39.00 ± 1.15	19.01 ± 0.89	34.63 ± 0.55	33.05 ± 0.59
	100%	22.55 ± 0.43	33.51 ± 0.37	43.61 ± 0.49	40.86 ± 0.33	19.69 ± 0.40	36.10 ± 0.59	34.10 ± 0.37

S4: Inhibition zone diameters produced by *R. canina* pseudo-fruit (WF)–derived extracts at different concentrations (10, 20, 50, and 100%) against pathogenic isolates and reference strains. Notes: Values are expressed as the mean of three replicates; For each disk content, yellow indicates the largest inhibition-zone diameter, light blue the second largest, and red the third largest
