# Supplementary material for: Evaluation of Plant-Guided Strategies Against Clinical Multidrug-Resistant Pathogens: Preliminary Phytochemical Screening, Antioxidant Capacity, and Antibacterial/Antibiofilm Activity of Rosa canina and Colchicum autumnale Extracts
Source: Antibiotics (Basel). 2026 May 18;15(5):508. doi: 10.3390/antibiotics15050508 (PMC13203422; doi:10.3390/antibiotics15050508)
Supplement: Supplementary file 1 [file antibiotics-15-00508-s001.zip › S3.pdf]

ID Pathogen/ Reference strain	Disc content	Colchicum autumnale flower fractions (CA)						
		A	E40	E60	ENZ	n-H	EtOAc	n-BuOH
S. aureus 78	10%	6.73 ± 0.74	10.34 ± 0.65	16.21 ± 0.68	17.97 ± 0 .37	6.36 ± 0.21	8.35 ± 0.52	13.07 ± 0.07
	20%	7.22 ± 0.2	12.45 ± 0.26	22.79 ± 0.60	42.04 ± 0.72	7.22 ± 0.20	15.15 ± 0.25	17.37 ± 0.25
	50%	13.83 ± 0.29	15.81 ± 0.67	53.66 ± 0.24	64.80 ± 0.21	7.43 ± 0.35	20.85 ± 0.67	23.75 ± 1.51
	100%	18.46 ± 0.71	17.43 ± 0.11	59.02 ± 0.83	67.53 ± 0.20	9.19 ± 0.10	28.76 ± 0.56	43.68 ± 0.47
K. pneumoniae 18	10%	6.37 ± 0.44	8.21 ± 0.51	13.46 ± 0.38	10.63 ± 0.17	6.01 ± 0.02	7.33 ± 0.39	12.46 ± 0.35
	20%	6.91 ± 0.02	11.51 ± 0.37	14.06 ± 0.19	15.63 ± 0.14	6.05 ± 0.05	10.77 ± 0.74	14.18 ± 0.72
	50%	9.62 ± 0.46	14.18 ± 0.77	28.56 ± 0.66	20.24 ± 0.98	7.47 ± 0.39	14.01 ± 0.31	26.47 ± 0.33
	100%	10.80 ± 0.27	15.53 ± 0.59	42.98 ± 1.5	29.23 ± 0.54	8.02 ± 0.15	18.24 ± 0.42	33.08 ± 0.99
K.pneumoniae 94	10%	7.19 ± 0.02	11.81 ± 0.46	12.49 ± 0.44	10.69 ± 0.41	6.06 ± 0.35	8.65 ± 0.35	12.89 ± 0.28
	20%	10.34 ± 0.22	15.94 ± 0.59	18.98 ± 0.83	20.61 ± 0.57	7.06 ± 0.16	12.51 ± 0.39	10.28 ± 1.04
	50%	14.14 ± 0.95	22.87 ± 0.96	26.62 ± 0.85	23.83 ± 0.86	10.00 ± 0.22	26.33 ± 0.13	30.29 ± 0.24
	100%	17.39 ± 0.39	28.14 ±1.56	49.27 ± 0.94	38.69 ± 0.50	10.89 ± 0.04	28.20 ± 0.98	30.07 ± 0.73
K.pneumoniae 109	10%	9.86 ± 0.13	11.32 ± 0.92	14.04 ± 0.26	12.17 ± 0.62	7.10 ± 0.05	10.08 ± 0.47	12.22 ± 0.93
	20%	12.49 ± 0.68	12.03 ± 0.37	21.17 ± 0.95	16.13 ± 0.09	8.61 ± 0.25	12.07 ± 0.34	15.57 ± 0.53
	50%	17.26 ± 0.54	15.96 ± 0.74	43.57 ± 0.37	27.24 ± 0.29	18.74 ± 0.24	30.84 ± 0.09	25.70 ± 0.50
	100%	24.97 ± 1.17	29.03 ± 0.24	61.85 ± 0.59	34.15 ± 0.98	18.92 ± 0.07	33.53 ± 0.49	42.55 ± 0.17
K.pneumoniae 181	10%	6.02 ± 0.01	7.29 ± 0.24	13.31 ± 0.45	10.27 ± 0.36	6.00 ± 0.01	7.59 ± 0.49	10.34 ± 0.94
	20%	6.08 ± 0.01	7.61 ± 0.58	17.51± 0.49	12.49 ± 0.27	6.09 ± 0.03	10.11 ± 0.11	11.48 ± 0.44
	50%	7.13 ± 0.07	12.7 ± 0.43	18.02 ± 0.19	17.72 ± 0.52	7.09 ± 0.50	13.41 ± 0.43	18.05 ± 0.15
	100%	7.82 ± 0.09	18.52 ± 0.35	27.15 ± 1.67	19.24 ± 0.54	9.37 ± 0.29	19.84 ± 0.25	20.78 ± 0.24
K. pneumoniae 328	10%	10.37 ± 0.45	11.36 ± 1.10	15.84 ± 0.90	13.03 ± 0.49	7.55 ± 0.45	10.68 ± 0.52	11.50 ± 0.37
	20%	10.34 ± 0.27	15.38 ± 0.09	22.12 ± 0.44	19.77 ± 1.23	7.89 ± 0.36	12.75 ± 0.28	13.65 ± 0.08
	50%	13.01 ± 0.77	26.52 ± 0.44	38.29 ± 1.36	21.52 ± 0.12	11.5 ± 0.30	15.51 ± 0.68	24.93 ± 0.71
	100%	21.34 ± 0.26	28.42 ± 0.33	51.34 ± 1.36	37.46 ± 1.21	16.23 ± 0.12	22.07 ± 1.51	40.11 ± 0.71
P. Aeruginosa 40	10%	6.38 ± 0.23	10.67± 0.38	15.50 ± 0.50	11.93 ± 0.40	7.35 ± 0.27	10.85 ± 0.49	12.40 ± 0.31
	20%	7.57 ± 0.36	11.52 ± 0.29	22. fo ± 0.57	15.85 ± 1.01	7.85 ± 0.07	15.93 ± 0.18	20.75 ± 0.40
	50%	11.51± 0.40	17.18 ± 0.72	34.39 ± 0.51	24.67 ± 0.18	11.41 ± 0.27	27.51 ± 1.22	31.43 ± 0.04
	100%	14.13 ± 0.70	20.66 ± 0.55	48.45 ± 0.27	33.52 ± 0.39	21.02 ±0.13	34.65 ± 0.70	44.83 ± 0.83
P. aeruginosa 309	10%	6.05 ± 0.03	10.22 ±0.52	12.11 ± 0.12	10.74 ± 0.35	6.19 ± 0.33	10.93 ± 1.02	13.38 ± 0.38
	20%	6.56 ± 0.32	11.82 ± 0.26	19.10 ± 0.31	13.36 ± 0.53	6.70 ± 0.25	13.89 ± 0.63	19.87 ± 0.04
	50%	12.09 ± 1.10	15.13 ± 0.38	30.53 ± 1.65	23.46 ± 0.39	8.33 ± 0.46	26.27 ± 9.17	32.54 ± 0.42
	100%	12.38 ± 0.21	17.78 ± 0.40	41.32 ± 0.34	28.78 ± 0.72	10.78 ± 0.46	33.48 ± 0.44	42.99 ± 0.73
P. aeruginosa ATCC 27853	10%	11.06 ± 0.43	11.65 ± 0.36	22.72 ± 0.82	15.92 ± 0.18	10.72 ± 0.23	11.57 ± 0.50	11.28 ± 0.52
	20%	13.42 ± 0.55	15.11 ± 0.42	27.55 ± 0.53	20.63 ± 0.26	13.91 ± 0.54	16.57 ± 0.60	20.57 ± 0.76
	50%	22.49 ± 0.57	25.18 ± 0.25	46.30 ± 0.79	34.23 ± 1.63	19.43 ± 0.43	29.95 ± 1.39	41.76 ± 1.44
	100%	22.64 ± 0.45	37.05 ± 1.39	61.21 ± 0.44	53.77 ± 1.35	21.45 ± 1.08	50.46 ± 1.25	55.46 ± 0.58
S. aureus ATCC 25923	10%	7.16 ± 0.50	10.44 ± 0.38	33.13± 1.49	17.27 ± 0.38	6.73 ± 0.20	7.36 ± 0.19	12.93 ± 0.72
	20%	7.22 ± 0.20	13.78 ± 0.35	20.07 ± 0.39	25.63 ± 0.34	7.38 ± 0.26	14.11 ± 0.19	18.52 ± 0.30
	50%	14.57 ± 0.24	21.61 ± 0.40	31.84 ± 1.74	40.80 ± 0.15	7.97 ± 0.03	21.27 ± 0.73	23.27 ± 1.94
	100%	19.79 ± 0.51	28.60 ± 0.42	50.62 ± 0.53	58.13 ± 0.56	10.32 ± 0.19	36.99 ± 1.67	49.41 ± 0.42
K. quasipneumoniae ATCC 700603	10%	11.03 ± 0.11	12.75 ± 0.15	15.59 ± 0.62	17.18 ± 0.63	7.83 ± 0.25	10.61± 0.33	15.56 ± 0.59
	20%	12.87 ± 0.28	15.46 ± 0.43	18.91 ± 0.39	16.51 ± 0.40	9.86 ± 0.29	17.77± 0.19	22.97 ± 33.13
	50%	25.82 ± 0.49	31.06 ± 0.51	48.17 ± 2.44	41.44 ± 0.97	16.09 ± 0.27	24.02 ± 0.47	33.13± 1.49
	100%	31.06 ± 0.17	50.53± 1.10	48.22 ± 0.75	52.59 ± 0.35	21.51 ± 0.54	50.46 ± 1.25	54.37 ± 1.46

S3: Inhibition zone diameters produced by *C. autumnale* flower–derived extracts at different concentrations (10, 20, 50, and 100%) against pathogenic isolates and reference strains. Notes: Values are expressed as the mean of three replicates; For each disk content, yellow indicates the largest inhibition-zone diameter, light blue the second largest, and red the third largest
