# Supplementary material for: Evaluation of Plant-Guided Strategies Against Clinical Multidrug-Resistant Pathogens: Preliminary Phytochemical Screening, Antioxidant Capacity, and Antibacterial/Antibiofilm Activity of Rosa canina and Colchicum autumnale Extracts
Source: Antibiotics (Basel). 2026 May 18;15(5):508. doi: 10.3390/antibiotics15050508 (PMC13203422; doi:10.3390/antibiotics15050508)
Supplement: Supplementary file 1 [file antibiotics-15-00508-s001.zip › S2.pdf]

Concentration (μg/mL)	*Colchicum autumnale* flower fractions (CA)							
	n-H	EtOAc	n-BuOH	A	E40	E60	ENZ	p-value
25	0.027 ± 0.006^{c}	0.217 ± 0.006^{a}	0.233 ± 0.012^{a}	0.117 ± 0.006^{c}	0.177 ± 0.006^{b}	0.203 ± 0.006^{b}	0.217 ± 0.012^{a}	0.004581
50	0.043 ± 0.006^{f}	0.420 ± 0.010^{b}	0.483 ± 0.006^{a}	0.187 ± 0.006^{e}	0.323 ± 0.006^{d}	0.353 ± 0.006^{c}	0.407 ± 0.006^{b}	0.003245
100	0.060 ± 0.000^{g}	0.847 ± 0.012^{b}	0.900 ± 0.010^{a}	0.297 ± 0.012^{f}	0.620 ± 0.010^{e}	0.690 ± 0.000^{d}	0.757 ± 0.006^{c}	0.003033
150	0.080 ± 0.010^{g}	1.157 ± 0.006^{a}	0.217 ± 0.012^{f}	0.440 ± 0.000^{e}	0.903 ± 0.012^{d}	1.007 ± 0.006^{c}	1.047 ± 0.012^{b}	0.003066
200	0.337 ± 0.401^{d}	1.400 ± 0.010^{a}	1.483 ± 0.006^{a}	0.550 ± 0.010^{d}	1.107 ± 0.006^{c}	1.247 ± 0.006^{bc}	1.307 ± 0.006^{b}	0.003575
250	0.117 ± 0.012^{b}	1.088 ± 0.808^{a}	1.603 ± 0.012^{a}	0.650 ± 0.010^{b}	1.250 ± 0.010^{b}	1.447 ± 0.012^{a}	1.503 ± 0.006^{a}	0.01103
Rosa canina pseudo-fruit fractions (WF)								
25	0.083 ± 0.006^{d}	0.247 ± 0.012^{b}	0.230 ± 0.000^{cd}	0.233 ± 0.006^{c}	0.253 ± 0.006^{b}	0.267 ± $0.012a^{b}$	0.307 ± 0.006^{a}	0.004462
50	0.120 ± 0.010^{d}	0.497 ± 0.006^{c}	0.483 ± 0.012^{c}	0.453 ± 0.012^{d}	0.520 ± 0.010^{b}	0.537 ± 0.012^{b}	0.583 ± 0.006^{a}	0.003399
100	0.187 ± 0.006^{d}	0.943 ± 0.006^{b}	0.920 ± 0.010^{c}	0.880 ± 0.010cd	1.003 ± 0.012^{b}	4.153 ± 5.410^{a}	1.100 ± 0.010^{a}	0.003575
150	0.243 ± 0.006^{g}	1.297 ± 0.012^{d}	1.247 ± 0.006^{e}	1.200 ± 0.010^{f}	1.347 ± 0.006^{c}	1.380 ± 0.010^{b}	1.483 ± 0.012^{a}	0.003131
200	0.293 ± 0.006^{g}	1.553 ± 0.012^{d}	1.497 ± 0.012^{e}	1.440 ± 0.000f	1.603 ± 0.006^{c}	1.647 ± 0.012^{b}	1.750 ± 0.010^{a}	0.003066
250	0.350 ± 0.010^{g}	1.710 ± 0.000^{d}	1.647 ± 0.012^{e}	1.603 ± 0.006^{f}	1.753 ± 0.012^{c}	1.803 ± 0.006^{b}	1.900 ± 0.010^{a}	0.003082
	Reference	25 μg/mL	50 μg/mL	100 μg/mL	150 μg/mL	200 μg/mL	250 μg/mL	
	Gallic acid	0.187 ± 0.006	0.423 ± 0.006	1.817 ± 0.006	1.857 ± 0.006	1.867 ± 0.006	1.887 ± 0.006	
	Ascorbic acid	0.180 ± 0.000	0.423 ± 0.006	0.683 ± 0.006	1.123 ± 0.006	1.660 ± 0.000	1.883 ± 0.006	

S2: Ferric reducing antioxidant power (FRAP) of solvent-partitioned fractions from *C. autumnale* flowers (CA) and *R. canina* pseudo-fruits (WF), expressed as absorbance values across the tested concentration range (mean ± SD, n = 3). Notes: Within each plant source and for each concentration (row), mean values with different superscript letters differ significantly among extracts (Kruskal–Wallis test, $p < 0.05$; Tukey-type multiple-comparisons procedure applied to rank-transformed absorbance values)
